# Unlocking Unexpected Charge Transfer Pathways in Interconnected Nanostructures

**DOI:** 10.1021/acsami.4c12205

**Published:** 2024-10-15

**Authors:** Kenan Elibol, Marko Burghard, Tobias Heil, Peter A. van Aken

**Affiliations:** Max Planck Institute for Solid State Research, Heisenbergstr. 1, 70569 Stuttgart, Germany

**Keywords:** charge transfer plasmon, electron energy-loss spectroscopy, nanofabrication, graphene, monochromated scanning transmission electron microscopy

## Abstract

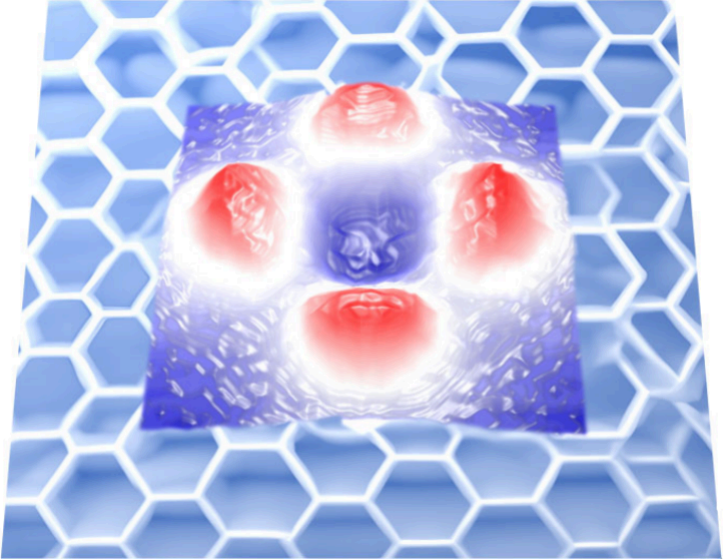

Accurate control of charge transfer pathways is critical to unlocking the full potential of charge transfer plasmons (CTPs) and exploring their diverse applications. We show that the intentional manipulation of junctions in Al nanocrosses on graphene induces asymmetry, unlocking unexpected charge transfer pathways and facilitating the generation of coupled resonators. The junction asymmetry, which is induced by nanotrench formation facilitated by focused electron beam irradiation, provides a versatile means to achieve precise and controlled interconnect manipulation. We find that tuning the nanotrench dimensions in nanocrosses allows for the tailored modulation of the charge transfer speed and the energies of CTPs. Furthermore, CTPs excited in our experimental nanocrosses, featuring nanotrenches, exhibit weak coupling. This crucial insight underscores the importance of controlled trench formation in unlocking various functionalities of CTPs for use in sensing, catalysis, and energy conversion applications. The controlled manipulation of interconnects in Al nanocrosses thus emerges as a promising avenue for advancing the device performance in these fields.

## Introduction

The establishment of electromagnetic coupling arises when two plasmonic nanoparticles (NPs) are brought into close proximity, as extensively studied.^1,2^ In scenarios involving direct charge transfer or tunneling between NPs, the system manifests a low-energy charge transfer plasmon (CTP) complementing the well-known localized surface plasmon resonances (LSPRs) that facilitate robust light absorption and scattering at specific frequencies.^2−11^ While LSPRs primarily hinge on the individual NP’s dimensions and composition, the dynamics of CTPs are intricately influenced by the interaction between two or more distinct NPs.^2,9,12^ The CTPs are of great interest because of their potential applications in diverse fields such as sensing, catalysis, and molecular electronics.^9,13,14^ Given that nanostructures interconnected by conductive bridges serve as fundamental units in constructing optoelectronic devices,^15−18^ the precise control of charge transfer pathways is of paramount importance for exploring the diverse potential applications of CTPs. Notably, despite the critical role of charge transfer in these nanostructures, the precise manipulation of charge transfer pathways in patterned plasmonic systems has hitherto remained an unmet challenge. This knowledge gap underscores the necessity for innovative strategies in order to unlock the full potential of interconnected plasmonic nanostructures, paving the way for groundbreaking advancements in the design and functionality of optoelectronic devices.

CTPs have undergone extensive investigation across various bridged nanostructures, incorporating diverse plasmonic materials.^9,12,14,19−21^ However, a critical gap persists in our understanding, as the influence of junction asymmetry on CTP properties remains unexplored. Unlike gold and silver, which exhibit plasmon losses primarily in the visible region,^22,23^ aluminum (Al), an earth-abundant material, demonstrates applicability across both ultraviolet (UV) and visible regions of the spectrum.^24−28^ However, Al exhibits limited support for plasmons in the visible spectral range due to its interband transition around 800 nm.^29,30^ As a result, its plasmonic performance is particularly weak in the red region of the visible spectrum.^31−34^ Despite the challenges of substrate-induced red-shift and mode-mixing affecting the LSPRs and CTPs of these materials, our work shows that these can be overcome by monolayer graphene used as a template. Monolayer graphene, utilized for the synthesis and integration of metal NPs,^35−37^ proves to be an exceptional substrate, mitigating the aforementioned issues.^38,39^

In this study, utilizing electron-beam lithography, we systematically fabricate Al nanocrosses consisting of four Al nanoprisms positioned on monolayer graphene membranes. The junction asymmetry, a pivotal aspect of CTP modulation, is generated with precision via focused electron-beam irradiation creating nanotrenches. We explore the impact of nanotrench length and width on the detected CTP modes. In contrast to optical methods, which cannot access dark plasmon modes,^40^ our approach for the detection and visualization of CTPs employs electron energy-loss spectroscopy (EELS) within a monochromated scanning transmission electron microscope (STEM). This methodology allows for the comprehensive mapping of all plasmon modes, achieving both high spatial and spectral resolution.^8,39,41−45^ The fidelity of our observations is substantiated through boundary-element method (BEM) simulations and LC circuit modeling. Furthermore, our investigations are bolstered by the implementation of coupled oscillator model (COM) simulations of EEL spectra, offering an exploration of the interaction strength between distinct CTP resonances within nanocrosses featuring nanotrenches.

## Results and Discussion

### Tunable CTPs in Al Nanocrosses Integrated on Graphene

**Figure 1:**
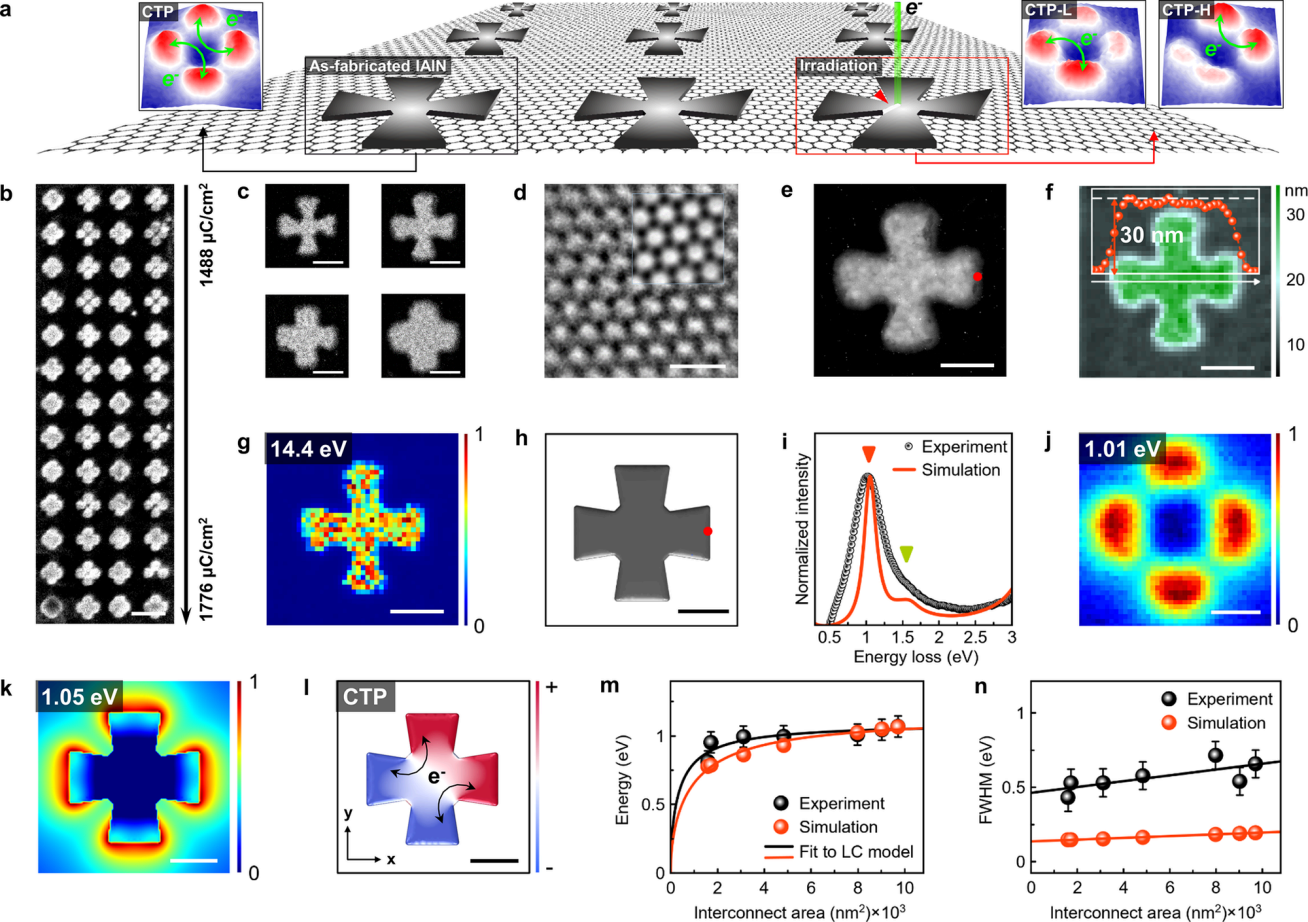
CTPs of Al nanocrosses fabricated on suspended monolayer graphene. (a) Schematic of Al nanocrosses involving four nanoprisms on suspended graphene. The structures shown within the black and red frames represent the as-fabricated and electron-beam-irradiated Al nanocrosses, respectively. Conductive junctions within nanocrosses are selectively modified by oxidation and focused electron beam irradiation to manipulate charge transfer modes. (b) Scanning electron microscopy (SEM) image illustrating Al nanocrosses fabricated on graphene affixed to a copper foil. The nanocrosses in (b) are patterned at different beam doses varied from 1488 to 1776 μC/cm^2^. (c) High-angle annular dark field (HAADF) images of nanocrosses, showing varying junction areas on a graphene membrane. (d) High-resolution transmission electron microscopy (HRTEM) image of graphene supporting Al nanocrosses. A simulated HRTEM image of graphene is superimposed on the experimental image for comparison (see blue-colored frame). (e) Close-up HAADF image of Al nanocrosses with a uniform junction. (f) Thickness map of the nanocross structure in (e). The inset displays the line profile along the white line on the thickness map. (g) VP map corresponding to the structure in (e). (h) Model employed in BEM simulations. (i) Experimental and simulated EEL spectra obtained at specific locations marked by red dots on the HAADF image in (e) and the model in (h). Red and green arrows denote the CTP resonance and IBT of Al, respectively. (j, k) Experimental and simulated EELS maps generated at ω_CTP_. (l) Computed eigenmode corresponding to the CTP resonance in (k). (m) Experimental and simulated ω_CTP_ obtained from Al nanocrosses with different interconnect areas. Solid black and red lines represent fitting to the LC circuit model. (n) Line widths of experimental and simulated CTPs as shown in (m). Solid black and red lines represent fitting to a linear function. Error bars in (m) and (n) indicate the standard deviation. The scale bars are 500 nm (b), 0.5 nm (d), and 100 nm (c, e–h, j–l).

We initially investigated the as-fabricated Al nanocrosses (Figure 1a) to elucidate the influence of junction dimensions on CTP properties. Arrays of Al nanocrosses featuring varying junction areas are fabricated on monolayer graphene membranes by electron-beam lithography (Figure 1b–e). The details of the nanofabrication process are described in the Methods section. The thickness of the nanocrosses fabricated on graphene is measured to be 30 nm (Figure 1f). Here, the suspended graphene is used to mitigate mode mixing and red-shifts of plasmon resonances in nanocrosses (Note S1 and Figure S1). As the volume plasmons (VPs) oscillate within the bulk, EELS maps of the VP unveil the morphology of metallic Al underlying the oxide layer (Figure 1g).^46,47^ It has been reported that the volume plasmon (VP) energy of aluminum (Al) can be altered by varying its temperature.^48−51^ When Al is heated to its melting temperature (∼660 °C), its VP energy, which is approximately 15 eV at room temperature, shifts to 14.2 eV.^49,50^ The reduction in VP energy is attributed to the thermal expansion of the Al volume, which lowers the free electron density. During our EELS measurements, where the sample is heated to 400 °C in a vacuum to remove hydrocarbon contaminants, we can expect a similar reduction in the VP energy of Al nanocrosses. Unlike the calculated VP energy (Figure S2), we observe a higher red-shift in VP energy at 400 °C. Since strain can influence the physical properties of metal clusters on graphene,^52,53^ the combined effects of thermal annealing and strain could well explain the reduced VP energy of 14.4 eV in Al nanocrosses fabricated on graphene.

To discern the plasmon resonances in Al nanocrosses with uniform junctions, we construct a BEM model (Figure 1h) mimicking the experimental structure in Figure 1e. Both experimental and simulated EEL spectra, obtained from the right edge of nanocrosses in Figure 1e and the corresponding BEM model in Figure 1h, exhibit a distinct resonance around 1 eV alongside the interband transition (IBT) of Al at 1.5 eV (Figure 1i).^54^ To identify the plasmon resonance marked by a red triangle in Figure 1i, spatially resolved EELS maps were extracted at specified energies (Figure 1j,k). Although the EELS maps offer partial elucidation, an eigenmode analysis (Figure 1l) is employed to implicitly discern the plasmon modes detected in the EEL spectra. The intense peak marked with a red triangle in Figure 1i is identified as a CTP mode, corresponding to an λ/2 resonance when considering antenna theory for plasmon mode identification.

Further clarification of the CTP resonances in Al nanocrosses is achieved through a coupled inductance–capacitance (LC) circuit model detailed in the Methods section (Note S2 and Figure S3). Electromagnetic simulations and LC circuit model analysis (Figure 1m) confirm that the sharp peak observed in the EELS measurements (peak marked with a red triangle in Figure 1i) corresponds to the CTP resonance. The sharper peak of CTP compared to other LSPR modes observed in EELS arises from the highly localized, less radiative, and strongly coupled nature of CTP modes, leading to lower energy dissipation and more confined plasmonic resonances (Figure S4). The plasmonic eigenmode suggests that electromagnetic coupling occurs only between two neighboring nanoprisms due to Coulomb screening between opposing nanoprisms (Figure 1l). Figure 1m demonstrates a blue-shift in the resonance energy of CTP (ω_CTP_) with increasing interconnecting area in nanocrosses. This is attributed to the reduction in kinetic inductance as the interconnect area expands.^38,55^ The minor discrepancies between the experimental and simulated data in Figure 1i,m result from variations in the dimensions of the experimental structure.

Distinct from CTPs, LSPRs of the nanocrosses in Figure 1e are observed at energies of >2 eV, significantly above the CTP energy (Note S3 and Figure S4). The lifetime of CTPs (τ_CTP_) is determined using the Heisenberg uncertainty relation given as
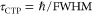
1where ℏ is Planck’s constant and FWHM is the line width of CTP.^47^ Since line widths are crucial for determining lifetimes, we present the FWHM’s of Al nanocrosses with varying interconnect areas (Figure 1n). The increase in the FWHM is attributed to the diminishing kinetic inductance. The large discrepancy between the experimental and simulated line widths is primarily due to the instrumental broadening of the peaks in EELS. As the interconnect areas increase, we observe a notable blue-shift in the λ resonance of Al nanocrosses as interconnect areas increase, while the corresponding 3λ/2 resonance remains unaffected (Figure S5 and Note S4). The existence of 3λ/2 and λ resonances shown in Figure S4 is further confirmed by LC circuit model analysis (Figure S6).

### Robust Control of Charge Transfer Pathways via Electron Beam Irradiation

**Figure 2:**
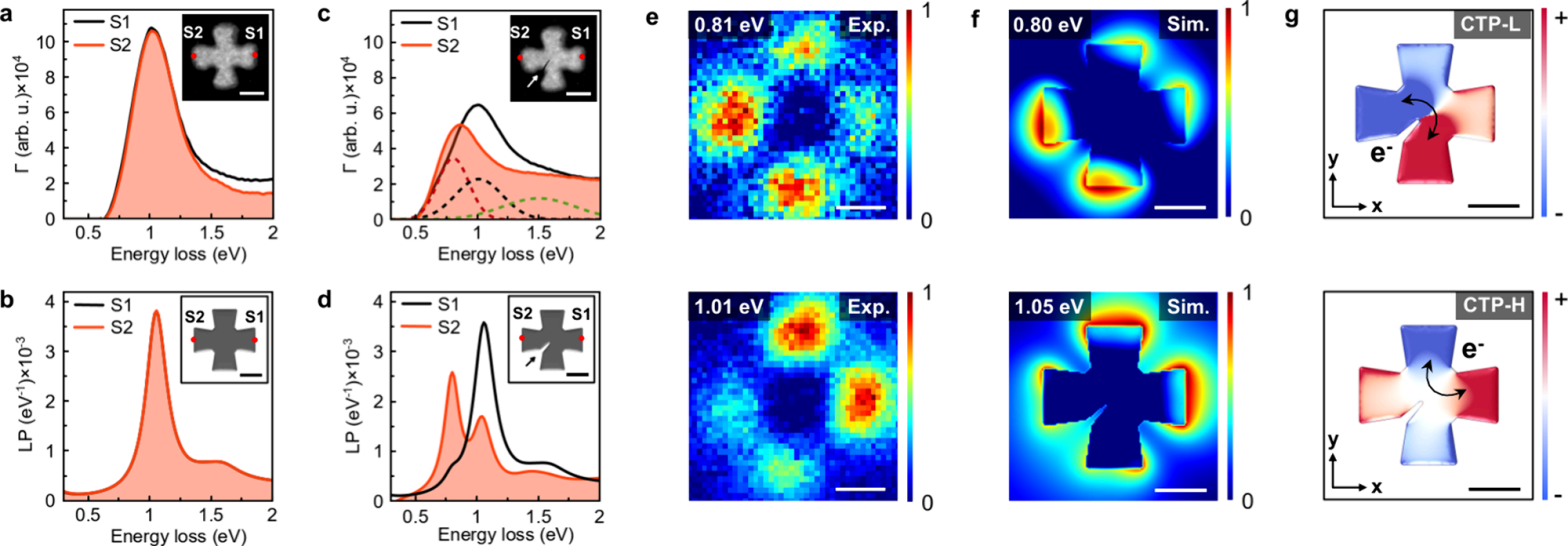
Tailoring charge transfer pathways through focused e-beam irradiation. (a, b) Experimental and simulated EEL spectra of an Al nanocross with a uniform interconnect. Spectra obtained from locations marked with red dots on the HAADF image and the model (inset of panels a and b). (c) EEL spectra obtained from both edges of the nanocross with an electron-beam-induced nanotrench. The dashed red, black, and green lines represent Gaussian curve fits for spectra obtained from the left edge of the nanocross. (d) BEM simulations of EEL spectra derived at marked positions on the model corresponding to the structure in (c). White and black arrows on the HAADF image and model indicate the nanotrenches created by the focused electron beam. (e) Spatially resolved EELS maps extracted at energies of 0.81 and 1.01 eV. (f) Simulated EEL spectra obtained at energies of 0.80 and 1.05 eV. (g) Calculated eigenmodes corresponding to the modes in (f). Scale bars are 100 nm (a–g).

To elucidate the behavior of CTPs in Al nanocrosses with distorted junctions, we used focused electron-beam irradiation within a STEM to manipulate nanocrosses (Figure 1a). This approach allows precise tailoring of CTPs via accurate control of nanotrenches dimensions. For nanocrosses with uniform junctions, EEL spectra acquired from opposite edges are identical, as also observed in the simulated EEL spectra (Figure 2a,b). The creation of an electron-beam-induced nanotrench within the nanocrosses results in a drastic change in the EEL spectra acquired from opposing edges (Figure 2c). The nanotrench is visible in both the HAADF image and the VP map (Figure 2c and Figure S7). The EEL spectra show that ω_CTP_, excited at the edge proximal to the nanotrench, is lower than ω_CTP_ obtained from the opposite edge. Deconvolved EEL spectra by curve fitting reveal two CTP modes resonating at low (ω_CTP-L_) and at slightly higher (ω_CTP-H_) energies in the nanocross with a nonuniform junction (Figure 2c). The presence of these two CTP resonances is confirmed in simulated EEL spectra derived from a structural model mimicking the experimental structure (Figure 2d).

EELS maps extracted at ω_CTP-L_ and ω_CTP-H_ suggest that electron transfer occurs predominantly between two adjacent nanoprisms (Figure 2e), which is confirmed by simulated EELS maps (Figure 2f). As shown in Figure 2g, computed eigenmodes reveal that CTP-L is activated during charge flow between nanoprisms partially separated by the nanotrench, while CTP-H is triggered by charge flow between nanoprisms located away from the nanotrench. The presence of nanotrenchs allows the excitation of two CTP modes without changing the resonance energies of the antibonding dipole LSPR and VP modes (Note S5 and Figure S8). The line width of a nanocross with a uniform junction is increased from 0.38 to 0.45 eV when an electron-beam-induced nanotrench is created within its junction. The experimental observations and BEM simulations together indicate that the line width of the CTP-L (0.30 eV) is shorter than the line width of the CTP-H (0.45 eV) in Al nanocrosses with nanotrenches (Note S6 and Figure S9). Our results suggest a transformation of nanocrosses, which initially behave as a single resonator, into two coupled resonators upon junction modification by nanotrench formation (Note S7 and Figure S10). Consistent results are observed for other nanocrosses with different junction areas (Note S8 and Figure S11).

In addition to the formation of nanotrenches that allow the formation of CTP-L and CTP-H modes, the partial oxidation of interconnects in Al nanocrosses can also induce the excitation of these modes (Note S9 and Figures S12–S15). Similar to the nanotrenches, the formation of an insulating oxide layer, which creates disconnected regions within the metallic interconnects of Al nanocrosses, induces junction asymmetry in nanocrosses. While oxidation induces distortion within Al nanocrosses, achieving precise control over the properties of CTPs remains a challenge (Note S9). Therefore, our primary focus is on the modulation of CTPs through the generation of nanotrenches.

**Figure 3:**
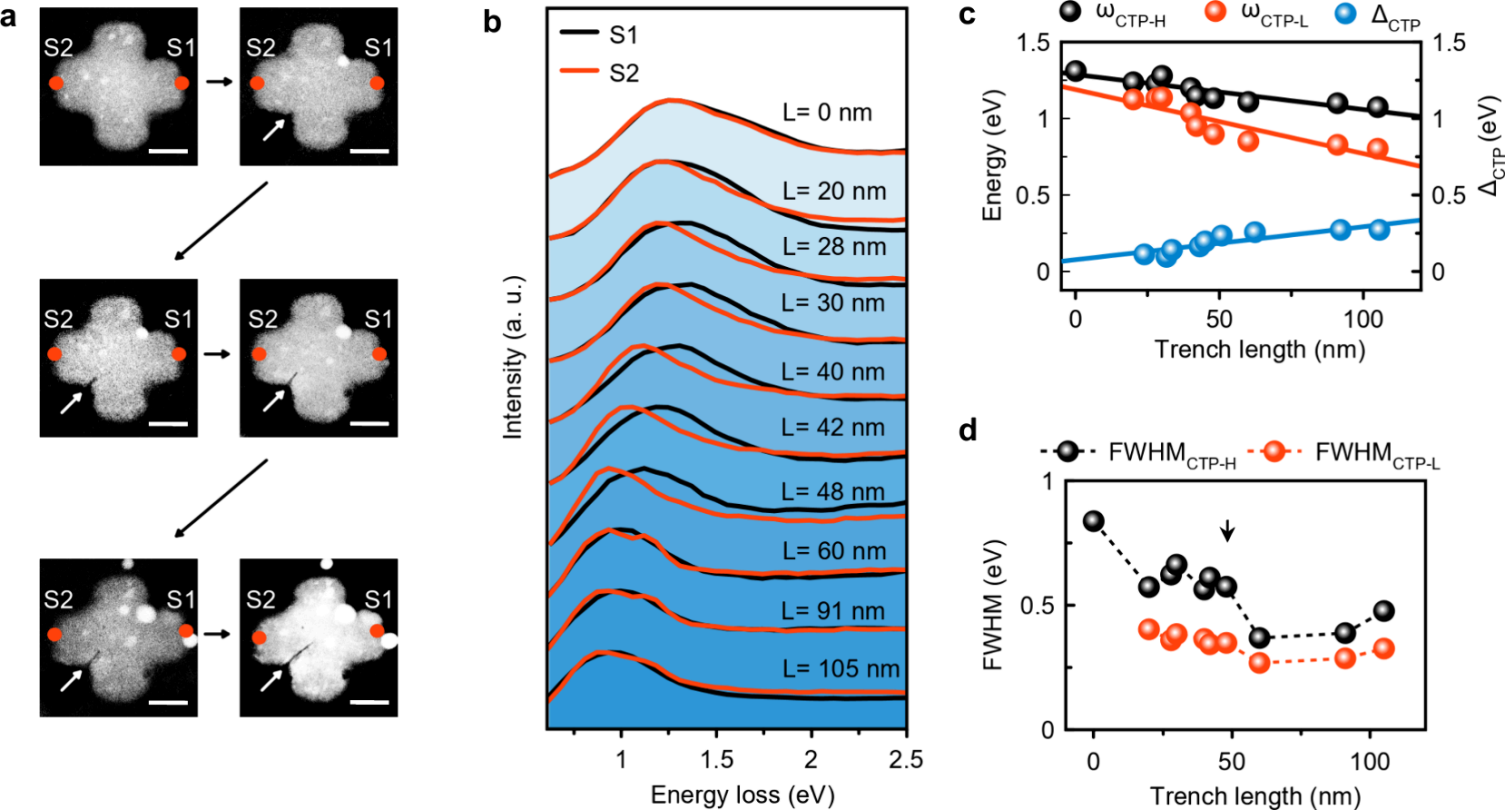
Effect of trench length on CTPs. (a) HAADF images of an Al nanocross before and after the introduction of nanotrenches of different lengths within its junction. White arrows indicate the location of the nanotrenches. (b) EEL spectra obtained from the nanocross with different trench lengths. Spectra obtained from locations marked with red dots on the HAADF images. (c, d) Evolution of ω_CTP-L_, ω_CTP-H_, Δ_CTP_, FWHM_CTP-L_, and FWHM_CTP-H_ as a function of trench length. The solid lines in (c) represent linear fits to the data, while the dashed lines in (d) serve as a guide for the eye. The black arrow on FWHM_CTP-L_ in (d) indicates the trench length (60 nm) that causes a significant change in FWHM_CTP-L_ and FWHM_CTP-H_. Scale bars are 100 nm (a).

To investigate the effect of trench length on the CTP resonances, we create electron-beam-induced nanotrenches of different lengths within the interconnect of a nanocross (Figure 3a). The trench sizes ranged from 0 to 105 nm. The white spots observed in the HAADF images represent hydrocarbon impurities fixed to the nanocross surface by the electron beam during nanotrench formation (Figure 3a). We note that hydrocarbon contamination generated during nanotrench formation may cause shifts and broadening of the CTP resonances. Figure 3b shows the EEL spectra acquired from opposite edges of the Al nanocross with and without nanotrenches. As can be seen in Figure 3c, the spectra obtained from both edges of the nanocrosses after nanotrench formation are not identical (Figure 3b). Both CTP modes derived from the EEL spectra deconvolved by Gaussian fitting show a red-shift with increasing trench size (Figure 3c and Figure S16). The Δ_CTP_, which represents the energy difference between ω_CTP-H_ and ω_CTP-L_, increases linearly with the trench length. Similar to the previous observation in Figure 2, FWHM_CTP-H_ is larger than FWHM_CTP-L_ (Figure 3d). Both FWHM_CTP-H_ and FWHM_CTP-L_ tend to decrease with an increasing trench length (Figure 3d). In particular, after a trench length of 48 nm, we observe a rapid decrease in the FWHM_CTP-H_ and FWHM_CTP-L_, which we attribute to the complete separation of CTP-L from CTP-H (Figure S16).

**Figure 4:**
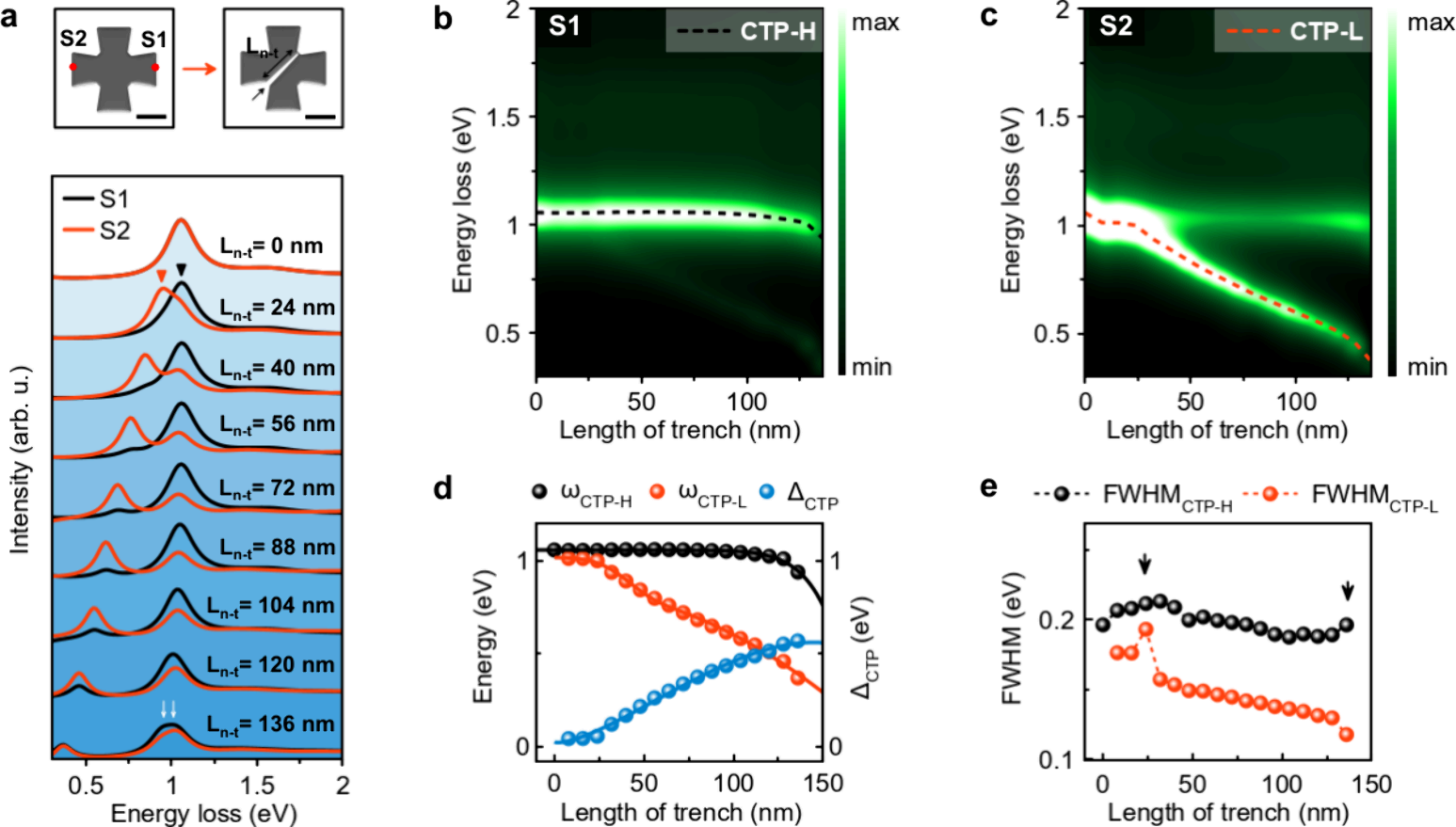
Computational study of the effect of trench size. (a) BEM simulations of EEL spectra derived at marked locations on Al nanocrosses with varying trench lengths, while the gap size is stationary. Red and black triangles indicate CTP-L and CTP-H modes in (a). (b, c) Counterplots of EEL spectra derived from opposite edges of Al nanocrosses with different trench lengths. (d, e) Evolution of ω_CTP-L_, ω_CTP-H_, Δ_CTP_, FWHM_CTP-L_, and FWHM_CTP-H_ as a function of trench length. Solid lines represent fits to the data, while dashed lines serve as guides for the eye. Black arrows on FWHM_CTP-L_ in (e) indicate trench lengths (24 and 136 nm) that cause a significant change in FWHM_CTP-L_. Scale bars are 100 nm (a).

To further investigate the effect of trench length on the CTPs in Al nanocrosses with distorted junctions, electromagnetic simulations are performed on a comparable nanocross structure (Figure 4). Similar to the experimental data shown in Figure 3b and Figure S16, the increasing trench length does not significantly alter ω_CTP-H_ but induces a red-shift in ω_CTP-L_ in the simulations (Figure 4a). However, when the trench length approaches the maximum of 136 nm, a new plasmon resonance close to CTP-H appears (white arrows on spectra shown in Figure 4a). The impact of the trench length on ω_CTP-H_ and ω_CTP-L_ is further evident in the counterplots of EEL spectra in Figure 4b,c. As observed in the experimental data (Figure 3c,d), Δ_CTP_ scales linearly with increasing trench length (Figure 4d), while both ω_CTP-L_ and ω_CTP-H_ values are reduced (Figure 4e). In the simulations, the decrease of ω_CTP-H_ is more visible when the trench length is longer than 120 nm while ω_CTP-H_ is found to be decrease more for the trench lengths longer than 40 nm. Similar to the observations in the experimental nanocross in Figure 3d, FWHM_CTP-H_ surpasses FWHM_CTP-L_ (Figure 4e). The rapid changes in FWHM_CTP-L_ (marked by the black arrow in Figure 4e) are attributed to the complete separation of CTP-L from CTP-H at a trench length of >24 nm and the formation of a new plasmon mode at a trench length of 136 nm. EELS simulations on Al nanocrosses with two nanotrenches show that the new mode appearing at the trench length of 136 nm is a low-energy dipolar gap mode (DGM), which is a bonding type (Note S10 and Figure S17). The DGM mode forms due to the near-field coupling between two closely spaced connected nanoprisms and vanishes when the separation distance between the two structures is >49 nm (Figure S17f,g). For Al nanocrosses with a single nanotrench, we find that it can also be induced when the junction width between two connected prisms is extremely small. When the joint width is sufficiently small, the system supports both CTP and DGM modes (Figure S17a,b). In contrast to single-trench nanocrosses (Figure 4a), ω_CTP-L_ excited in double-nanotrench Al nanocrosses decays nonlinearly due to the excitation and exponential reduction of the DGM resonance (Figure S17). While DGM modes form at Al nanocrosses without a bridge between nanoprism pairs, CTP-L forms only when all nanoprims are connected (Note S10).

**Figure 5:**
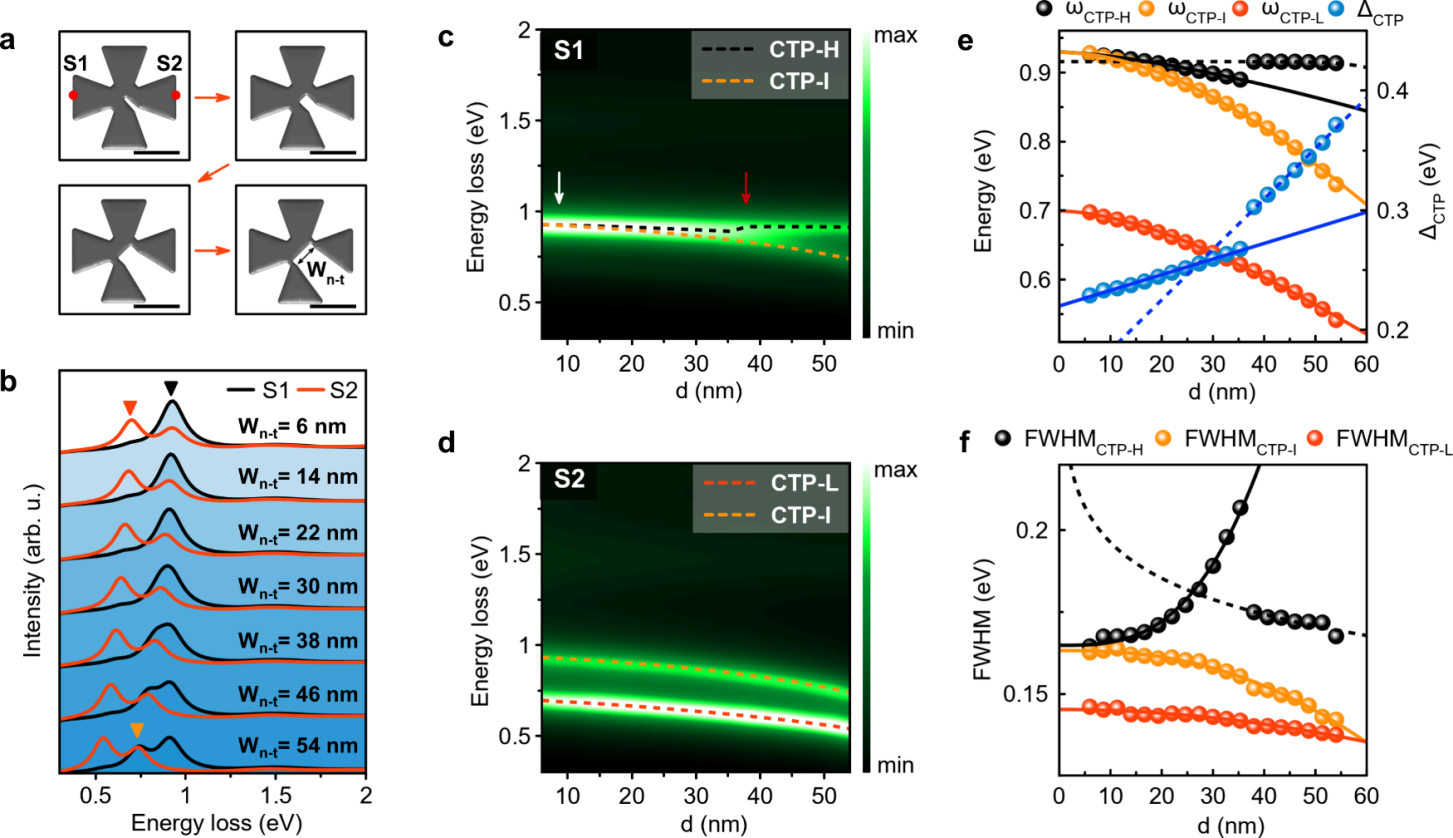
Emergence of an intermediate-energy CTP mode in Al nanocrosses with nanotrenches. (a) Illustrative models show nanocrosses with varying trench widths. (b) Simulated EEL spectra derived from different locations marked by red spots on the model in (a). Peaks marked by black, red, and orange triangles correspond to the CTPs excited at low, intermediate, and high energies, respectively. (c, d) Counterplot of simulated EEL spectra obtained from nanocrosses with different trench widths, each spectrum derived from locations marked with red spots in (a). White and red arrows in (c) pinpoint trench widths, leading to distinct changes in CTP modes. Trench width varies from 6 to 54 nm, while trench length is maintained at a constant 34 nm. (e) Variation of ω_CTP-L_, ω_CTP-I_, ω_CTP-H_, and Δ_CTP_ as a function of trench width. (f) Dependence of FWHM_CTP-L_, FWHM_CTP-I_, and FWHM_CTP-H_ on trench width. Black, orange, and red lines shown in (e) and (f) represent fitting curves to an exponential function. The scale bars are 100 nm (a).

To investigate the effect of the separation distance between neighboring nanoprisms within the Al nanocrosses, we conduct BEM simulations on Al nanocrosses with nanotrenches of different widths (Figure 5). Derived from distinct edges of nanocrosses denoted by red spots (Figure 5a), the EEL spectra reveal intriguing behaviors. Specifically, ω_CTP-L_, marked with a red triangle, undergoes a pronounced red-shift, while ω_CTP-H_, indicated by a black triangle, exhibits a minimal shift (Figure 5b). Analyzing EEL spectra from position S1 on nanocrosses (Figure 5a) reveals a subtle red-shift of ω_CTP-H_ with an incremental increase in trench width, reaching up to 38 nm (Figure 5c). Beyond this threshold, ω_CTP-H_ experiences a rapid increase to higher energies (see the red arrow in Figure 5c), remaining nearly constant for larger trench widths. Notably, a novel CTP mode, designated as CTP-I, emerges at intermediate energies for a trench width exceeding ∼9 nm (white arrow in Figure 5c). For the trench widths of <∼9 nm, CTP-I modes cannot be distinguished because the CTP-H mode, which has a higher energy loss probability, dominates the CTP-I mode. As shown above, trench length does not contribute to the formation of CTP-I. The excitation of CTP-I is validated through EELS maps and their corresponding eigenmodes (Note S11 and Figure S18). ω_CTP-L_ and ω_CTP-I_ excited at position S2 (Figure 5a) also exhibit nonlinear red-shifts with increasing trench width (Figure 5d). The difference between ω_CTP-I_ and ω_CTP-L_ remains relatively constant for trench widths of below 38 nm (Figure 5d). In Figure 5e, the rapid increase of ω_CTP-H_ is attributed to the nonlinear red-shift of ω_CTP-I_ for trench widths above 38 nm. When the trench width exceeds 38 nm, both ω_CTP-I_ and ω_CTP-H_ modes completely separate and can be clearly distinguished (Figure 5b,c). As a result, the line widths of the CTP-I and CTP-H modes can be accurately measured without their mutual interference at the trench widths of >38 nm. Unlike the consistent difference between ω_CTP-L_ and ω_CTP-I_, a linear enhancement is observed in Δ_CTP_, representing the difference between ω_CTP-L_ and ω_CTP-H_ (Figure 5e). The steeper slope of the linear curve is attributed to the faster decay in ω_CTP-I_ for trench widths ≥38 nm. Figure 5f reveals that FWHM_CTP-L_ and FWHM_CTP-I_ exponentially decrease with the trench width, while FWHM_CTP-H_ increases exponentially up to 38 nm, followed by a nonlinear decrease. Importantly, FWHM_CTP-L_ is shorter than those for FWHM_CTP-I_ and FWHM_CTP-H_. These findings underscore the crucial role of nanotrench formation in controlling the speed of charge flow in Al nanocrosses, thereby providing a versatile platform for tailored plasmonic functionalities.

### Weakly Interacting CTPs in Al Nanocrosses

To elucidate the interaction dynamics between CTP-L and CTP-H in Al nanocrosses featuring nanotrenches, we conduct COM simulations of EEL spectra (details in the Methods section). The plasmonic mode hybridization occurs explicitly when our nanocross systems fulfill the criterion in eq 2 or eq 3.^56^
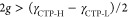
2
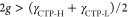
3where γ_CTP-H_ and γ_CTP-L_ represent the line widths of CTP-L and CTP-H modes, and *g* denotes the coupling strength. The stringent criterion in eq 3 requires a higher *g* for fulfillment.

**Figure 6:**
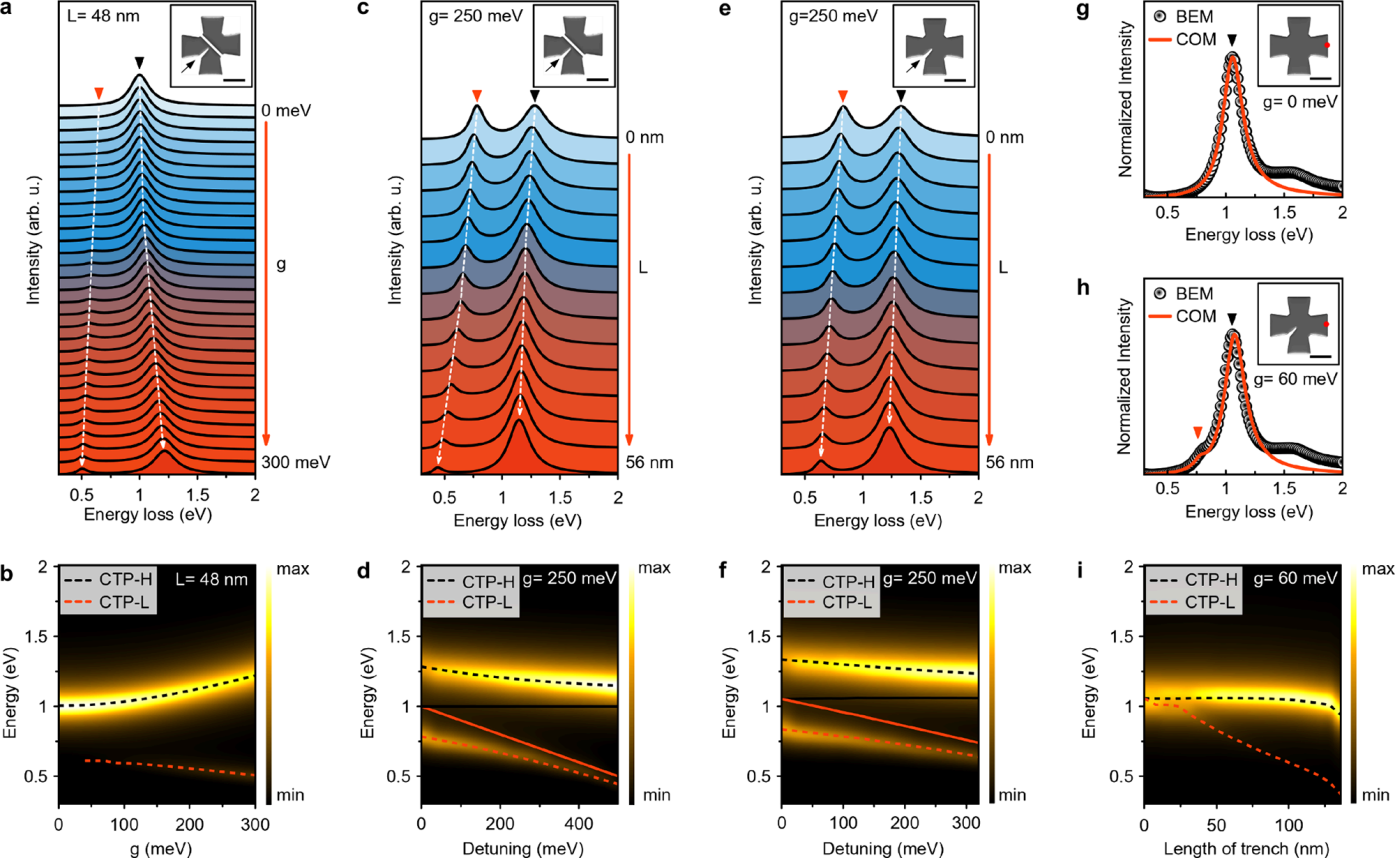
COM simulations of EEL spectra. (a) COM-simulated EEL spectra for a single-trenched Al nanocross with a trench length of 48 nm. (b) Counterplot of EEL spectra in (a) as the coupling strength varies from 0 to 300 meV. (c) EEL spectra simulated by COM (*g* = 250 meV) for single-trenched nanocrosses with trench lengths ranging from 0 to 56 nm. The dashed black and red lines show the energies of CTP-H and CTP-L. (d) Counterplot of EEL spectra in (c) in terms of detuning (ω_CTP-H_ – ω_CTP-L_). ω_CTP-H_ and ω_CTP-L_ are derived from BEM simulations of Al nanocrosses comprising two nanoprims (Figure S16a). The dashed black and red lines show the energies of CTP-H and CTP-L as a function of detuning, while the black and red solid lines are the uncoupled CTP energies for a single-trenched half-nanocross without and with a nanotrench. (e) COM simulations of EEL spectra for single-trenched nanocrosses with trench lengths varied from 0 to 56 nm when *g* = 250 nm. (f) Counterplot of EEL spectra in (e) depicting detuning (ω_CTP-H_ – ω_CTP-L_). ω_CTP-H_ and ω_CTP-L_ are based on BEM simulations of nanocrosses with four nanoprims (Figure S19b). The dashed black and red lines demonstrate the energies of CTP-H and CTP-L as a function of detuning, while the black and red solid lines are the uncoupled CTP energies for a single-trenched nanocross without and with a nanotrench. (g, h) BEM simulations of EEL spectra for a nanocross without and with a 48 nm long nanotrench. The spectra from marked positions on the models are fitted to the COM for *g* = 0 meV and *g* = 60 meV in (g) and (h), respectively. (i) Counterplot of COM simulations of EEL spectra for Al nanocrosses with trench lengths ranging from 0 to 136 nm when *g* = 60 meV. The red and black triangles indicate CTP-L and CTP-H in (a, c, e, g, h). The scale bars are 100 nm (a, c, e, g, h).

Considering that CTPs in single-trenched Al nanocrosses resonate at different energies and COM requires the energies and line widths of uncoupled oscillators, we bifurcate the nanocrosses and perform BEM simulations to ascertain the uncoupled CTP resonances and their line widths in the individual half-nanocrosses with and without a nanotrench (Note S12 and Figure S19a). COM simulations of EEL spectra shown in Figure 6a,b reveal that the plasmonic mode coupling is observed clearly when *g* ≥ 77 meV for a single-trenched nanocross with a trench length of 48 nm, meeting the conditions of both 2*g* and Rabi splitting exceeding (γ_CTP-H_ + γ_CTP-L_)/2 for *g* ≥ 77 meV. Rabi splitting is given as follows:
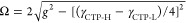
4The emergence of Ω becomes distinctly discernible when *g* ≥ 77 (Figure 6b), prompting an examination of plasmonic mode hybridization under detuning conditions (ω_CTP-H_ – ω_CTP-L_). This exploration involves varying the trench length at *g* = 250 meV (Figure 5c,d), revealing a minimum Ω of ∼499 meV at zero detuning (ω_CTP-H_ = ω_CTP-L_). It is pertinent to note the intentional inclusion of zero detuning, recognizing that Al nanocrosses lacking a nanotrench fail to produce CTP-L, thereby precluding zero detuning in these systems. In Figure 5e, COM simulations for nanocrosses with varied trench lengths unequivocally demonstrate two hybridized modes (CTP-H and CTP-L) at *g* = 250 meV. The resonance energies and line widths for CTP-H and CTP-L used in COM simulations are derived from BEM simulations conducted for single-trenched nanocrosses (Figure S19b). Strikingly, the congruence between the COM results depicted in Figure 6c,d and Figure 6e,f is noteworthy. As the nanocross with a uniform junction behaves like a single oscillator, the BEM simulations for *g* = 0 meV align with the COM simulations (Figure 6g). However, a single-trenched nanocross matches COM for *g* = 60 meV due to the interaction of CTP-H and CTP-L (Figure 6h). COM simulations also align well with experimental data for *g* = 0 and 60 meV (Note S13 and Figure S20). Furthermore, the weak interaction is reaffirmed in another single-trenched nanocross with a larger junction area (*g* = 10 meV) (Note S14 and Figure S21). Ultimately, COM simulations for Al nanocrosses with varying trench lengths align with BEM simulations (Figure 4b) for *g* = 60 meV (Figure 6i). Our results underscore that CTP-H and CTP-L excited at single-trenched Al nanocrosses exhibit weak interaction, placing these systems in the weak coupling regime, as evidenced by eq 5 in all experimental data.
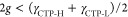
5

## Conclusions

This study establishes a profound understanding of charge transfer pathway modulation in Al nanocrosses, by strategically creating nanotrenches within their interconnects. These unique structures, generated through electron-beam irradiation processes, serve as a distinctive platform for tailoring the CTP properties. Electron beam irradiation provides control over nanotrench dimensions and CTP properties.

Our investigations revealed intriguing phenomena. CTP-L exhibits a significant red-shift, intensifying with increased trench length and width, accompanied by an implicit enhancement in its lifetime. In contrast, the resonance energy of CTP-H shows limited response to variations in trench length, but a subtle enhancement in lifetime is observed with an increase in trench length, while a reduction occurs with an increased trench width. Notably, widening the trench triggers the formation of a novel CTP-I resonance. Both CTP-L and CTP-I exhibit similar responses to variations in trench width, with CTP-I distinguished by a slightly higher resonance energy and a lower lifetime compared to CTP-L.

Furthermore, BEM simulations elucidate that low-energy DGMs minimally affect CTP-H but significantly contribute to the nonlinear decay of CTP-L resonances. Crucially, this decay follows a linear trend in single-trenched nanocrosses. COM simulations of EEL spectra confirm that asymmetry induced within nanocross junctions results in the formation of weakly coupled dual oscillators in otherwise uniform nanocross systems acting as a single resonator.

In summary, our findings offer valuable insights into the creation of new charge transfer pathways, crucial for tailoring plasmonic properties, and the formation of weakly coupled CTP resonances through the distortion of interconnects within complex bridged nanostructures. These results not only deepen our understanding of the fundamental principles governing plasmonic behavior but also open new avenues for the rational design of plasmonic systems with tailored functionalities, holding great promise for innovative applications in optoelectronics and beyond.

## Methods

### Nanofabrication

The fabrication process for the arrays of Al nanocrosses was executed on CVD-grown monolayer graphene situated on a Cu foil (Graphenea Inc.), employing electron-beam lithography. Prior to spin coating with PMMA, the graphene/Cu stack (approximately 4 × 4 mm^2^) underwent cleaning with acetone and isopropanol. Subsequently, it was affixed onto a flat Si substrate and coated with an 80–90 nm thick layer of PMMA (2.5% PMMA 950k in anisole) via a spinner operating at 6000 rpm (with an acceleration rate of 2000 rpm/s) for 35 s. Following completion of the resist spin coating, the sample was annealed at 160 °C for 4 min. Patterning of the resist was performed using a Raith eLine electron-beam lithography system, equipped with a 7.5 μm objective aperture and operated at an acceleration voltage of 15 kV. The working distance, beam current, and areal dose were set at 9 mm, 19.4 pA, and 1200 μC/cm^2^, respectively. The exposed resist was developed in a methyl isobutyl ketone (MIBK)/isopropyl alcohol (IPA) solution (3:1) at 0 °C for 30 s and dried using a nitrogen spray gun. Subsequent to the resist development, a 30 nm thick layer of Al (99.99% purity) was deposited onto the developed sample using a thermal evaporator (Univex 1) with a base pressure of ∼1.5 × 10^–6^ mbar. The deposition rate of Al was determined to be ∼2 Å/s, monitored by using a quartz crystal microbalance. The metal layer atop the PMMA was then lifted off by immersing the sample in *N*-methyl-2-pyrrolidone (NMP) heated at 60 °C for ∼30 min. Following this, the sample was rinsed with acetone and isopropanol. For the transfer of Al nanocrosses, we utilized an Au TEM grid featuring a holey carbon film (Quantifoil) covered with a 10 nm thick platinum film, deposited by using a sputter coater (Leica). The TEM grid on the sample was positioned using a home-designed micromanipulator. To enhance the adhesion between the Quantifoil and graphene, a drop of isopropanol was applied to the TEM grid on the sample. The Cu foil was subsequently etched away in a 10% ammonium persulfate (APS) solution applied for ^∼^3 h. Postetching, the nanocross/graphene stack attached to the TEM grid underwent rinsing with isopropanol. Both before and after the transfer process, the samples were imaged using an SEM instrument (Zeiss Gemini DSM 982 with a cold field-emission gun and an In-lens detector at an acceleration voltage of 5 kV). Prior to the STEM/EELS measurements, the samples were annealed at 300 °C for 15 min under ambient conditions to remove hydrocarbon adsorbates on the graphene surfaces.

### EELS and EDS Measurements

We conducted STEM and low-loss EELS measurements using the state-of-the-art sub-electronvolt–sub-angstrom microscope (Zeiss SESAM). This microscope, equipped with a Schottky field-emission gun, an electrostatic OMEGA-type monochromator, and a MANDOLINE filter boasting high dispersion and transmissivity, facilitated experiments with an energy resolution of 0.17 eV (Note S15 and Figure S22). Operating at an acceleration voltage of 200 kV, an energy dispersion of 0.015 eV/pixel, and an EELS collection semiangle of 0.7 mrad, we acquired EELS maps with a pixel dwell time of 0.5 s, summing over an energy window of 0.2 eV. Subsequent data processing involved a multivariate weighted principal component analysis (PCA) routine.^57^ The energies and line widths of the various plasmon modes were determined by peak fitting, applied to the EELS data after denoising by PCA. The samples were heated to 400 °C using an in-situ heating holder to prevent hydrocarbon contamination during EELS mapping. EDS measurements were performed under identical conditions at 80 kV.

### STEM and HRTEM Imaging

HRTEM imaging of graphene was achieved using a JEOL ARM200F FEG-STEM/TEM, featuring a cold field-emission gun, a postspecimen spherical aberration corrector (Cs), and a Gatan GIF Quantum ERS electron energy-loss spectrometer at an acceleration voltage of 80 kV at under-focus conditions. For STEM-HAADF images of Al nanocrosses, the JEOL ARM200F FEG-STEM/TEM and Zeiss SESAM were employed at an accelerating voltage of 200 kV.

### HRTEM Image Simulations

HRTEM image simulations were performed via the QSTEM software, incorporating parameters aligned with those used in the actual experiments. Simulations employed a spherical aberration coefficient of 1 μm, an accelerating voltage of 80 kV, and a defocus set at a wavelength of −2.5 nm.

### Electromagnetic Simulations

Electromagnetic simulations utilized the boundary element method (BEM) implemented in the Matlab toolbox MNPBEM.^58^ Nanostructure dimensions aligned with observed values, and an effective medium approach simplified computational complexity. The effective medium approach is a computational technique used to simplify the modeling of complex systems.^59^ When using an effective medium approach in BEM, the complex interactions between particles and their surrounding medium are taken into account by using effective optical constants for the surrounding medium or for inclusions within the nanoparticle. A Drude–Lorentz model approximated the complex dielectric function of aluminum. BEM simulations of EEL spectra used an electron-beam excitation with a beam energy of 200 keV, which were performed in the energy range of 0.3–4 eV. In the context of the quasi-static approximation, which is appropriate for small nanoparticles where retardation effects can be neglected, while still accounting for the full frequency dependence of the dielectric functions, the surface charges σ(*s*) at the particle boundary ∂V are calculated for the external potential ϕ_ext_(*r*) of the electron beam using the following equation:^60^
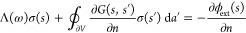
6In this context, *G*(*s*,*s*′) represents the Green’s function that connects the positions *s* and *s*′ on the particle boundary, while ∂/∂*n* denotes the derivative in the direction of the outer surface normal. In eq 6, the first term on the left-hand side depends solely on the material properties, expressed as
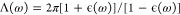
7where ϵ(ω) is the dielectric function of the metal. On the other hand, the second term is influenced only by the geometry of the nanoparticle. Thus, the plasmonic eigenmodes are calculated as follows:
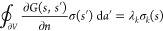
8In this case, λ_*k*_ represents the *k*th eigenvalue, and σ_*k*_(*s*) is its corresponding eigenfunction. The energies of the plasmonic eigenmodes are given as
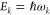
9These energies are determined by solving the
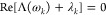
10

### LC Circuit Model

An equivalent circuit model for a coupled LC resonator is shown in Figure S3. The impedance (*Z*) of this coupled LC circuit is
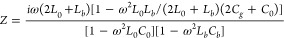
11where *L*_*b*_ and *C*_*b*_ are nanoinductors and nanocapacitors in the junctions, *C*_*g*_ is a nanocapacitor used to define gaps between NPs, and *L*_0_ and *C*_0_ are nanoinductors and nanocapacitors in nanoprims acting as LC tank oscillators. We applied the following assumptions to simply the model: (i)
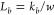
12where *k*_*b*_ is a fitting parameter involving the kinetic inductivity, length, and height of the junction. (ii) As the kinetic inductance dominates in the narrow junctions, we disregarded the geometric inductance. (iii) For the connected nanoprisms, *C*_*g*_ was assumed to be constant. The CTP resonances were found by solving the resonant conditions for the given LC circuit. The resonance frequency of a coupled resonator circuit is given as^61^
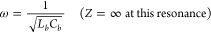
13This resonance fulfills the condition for a CTP mode excited at connected nanoprisms. Thus, the CTP resonance was obtained by fitting data to eq 14.
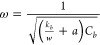
14where *k*_*b*_, *a*, and *C*_*b*_ are treated as fitting parameters, while *w* represents the junction width (Figure S3). The dark λ resonance is observed when
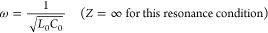
15Lastly, we describe the resonance condition of the 3λ/2 resonance as
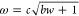
16where both *c* and *b* are used as fitting parameters.

### Electron Beam Manipulation

We achieved precise manipulation of interconnects within Al nanocrosses through focused electron-beam irradiation utilizing a JEOL ARM200F STEM/TEM equipped with a probe Cs corrector. The focused electron beam, accelerated at 200 kV, was directed onto the interconnects of Al nanocrosses for approximately 1 min, inducing knock-on damage that facilitated the controlled formation of nanoscale trenches. Given the presence of residual water and hydrocarbons in the samples, the electron beam acted as an etching agent, ionizing species and oxidizing the metallic Al.^62^ To ensure precise separation within the junction, the electron beam was strategically positioned at various locations on the interconnects of the Al nanocrosses. A probe size of 2*C*, corresponding to a beam diameter of 0.27 nm, was selected to provide a high beam current. The experiments were conducted with a beam current of 15 μA and a current density of 83 pA/cm^2^, ensuring optimal conditions for the precise and controlled manipulation of nanostructure junctions.

### Coupled-Oscillator Model Simulations of EEL Spectra

In our investigation, we employed a coupled oscillator model to describe the low- and high-energy CTP resonances excited in the Al nanocrosses. Within the framework of this model, the extinction at a frequency ω for two uncoupled damped harmonic oscillators is expressed as^63^
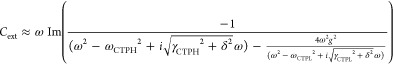
17where ω_CTPL_ and ω_CTPH_ represent the frequencies of CTP-L and CTP-H modes, while γ_CTPL_ and γ_CTPH_ denote the line widths of these modes. Here, the line widths of CTP resonances were obtained from the curve-fitting of EEL spectra. The line widths were accurately determined through the curve-fitting analysis of EEL spectra, with an instrumental broadening factor δ set at 80 meV. The coupling strength was estimated by fitting this model to the experimentally obtained EEL spectra, providing a quantitative measure of the interaction between the two oscillators in the system.

## Supporting Information Available

Additional details on substrate effect, LC circuit model, LSPRs, impact of junction area on CTPs and LSPRs of Al nanocrosses, effect of nanotrench on LSPR and VP, CTPs in Al nanocrosses with an e-beam-induced nanotrench, formation of two oscillators via junction distortion, CTP modes in Al nanocrosses deformed by focused e-beam irradiation, CTP modulation through interconnect oxidation, formation of DGM resonance, identification of CTP-I resonance, BEM simulations for Al nanocrosses with varying trench lengths, COM fitting to experimental EEL spectra, energy resolution; additional STEM, TEM, and EELS data and additional electromagnetic simulations (PDF)

## References

[ref1] Schuller J. A.; Barnard E. S.; Cai W.; Jun Y. C.; White J. S.; Brongersma M. L. Plasmonics for extreme light concentration and manipulation. Nat. Mater. 2010, 9 (3), 193–204. 10.1038/nmat2630. 20168343

[ref2] Tan S. F.; Wu L.; Yang J. K. W.; Bai P.; Bosman M.; Nijhuis C. A. Quantum Plasmon Resonances Controlled by Molecular Tunnel Junctions. Science 2014, 343 (6178), 1496–1499. 10.1126/science.1248797. 24675958

[ref3] Lerch S.; Reinhard B. M. Effect of interstitial palladium on plasmon-driven charge transfer in nanoparticle dimers. Nat. Commun. 2018, 9 (1), 1608 10.1038/s41467-018-04066-2. 29686266 PMC5913128

[ref4] Xiang L.; Palma J. L.; Bruot C.; Mujica V.; Ratner M. A.; Tao N. Intermediate tunnelling–hopping regime in DNA charge transport. Nat. Chem. 2015, 7 (3), 221–226. 10.1038/nchem.2183. 25698331

[ref5] Byers C. P.; Zhang H.; Swearer D. F.; Yorulmaz M.; Hoener B. S.; Huang D.; Hoggard A.; Chang W.-S.; Mulvaney P.; Ringe E.; et al. From tunable core-shell nanoparticles to plasmonic drawbridges: Active control of nanoparticle optical properties. Science Advances 2015, 1 (11), e1500988 10.1126/sciadv.1500988. 26665175 PMC4672758

[ref6] Esteban R.; Borisov A. G.; Nordlander P.; Aizpurua J. Bridging quantum and classical plasmonics with a quantum-corrected model. Nat. Commun. 2012, 3 (1), 825 10.1038/ncomms1806. 22569369

[ref7] Wu L.; Duan H.; Bai P.; Bosman M.; Yang J. K. W.; Li E. Fowler–Nordheim Tunneling Induced Charge Transfer Plasmons between Nearly Touching Nanoparticles. ACS Nano 2013, 7 (1), 707–716. 10.1021/nn304970v. 23215253

[ref8] Wiener A.; Duan H.; Bosman M.; Horsfield A. P.; Pendry J. B.; Yang J. K. W.; Maier S. A.; Fernández-Domínguez A. I. Electron-Energy Loss Study of Nonlocal Effects in Connected Plasmonic Nanoprisms. ACS Nano 2013, 7 (7), 6287–6296. 10.1021/nn402323t. 23782059

[ref9] Wen F.; Zhang Y.; Gottheim S.; King N. S.; Zhang Y.; Nordlander P.; Halas N. J. Charge Transfer Plasmons: Optical Frequency Conductances and Tunable Infrared Resonances. ACS Nano 2015, 9 (6), 6428–6435. 10.1021/acsnano.5b02087. 25986388

[ref10] Wang Y.; Yu J.; Mao Y.-F.; Chen J.; Wang S.; Chen H.-Z.; Zhang Y.; Wang S.-Y.; Chen X.; Li T.; et al. Stable, high-performance sodium-based plasmonic devices in the near infrared. Nature 2020, 581 (7809), 401–405. 10.1038/s41586-020-2306-9. 32461649

[ref11] Liu H.; Gage T. E.; Singh P.; Jaiswal A.; Schaller R. D.; Tang J.; Park S. T.; Gray S. K.; Arslan I. Visualization of Plasmonic Couplings Using Ultrafast Electron Microscopy. Nano Lett. 2021, 21 (13), 5842–5849. 10.1021/acs.nanolett.1c01824. 34153185

[ref12] Pérez-González O.; Zabala N.; Borisov A. G.; Halas N. J.; Nordlander P.; Aizpurua J. Optical Spectroscopy of Conductive Junctions in Plasmonic Cavities. Nano Lett. 2010, 10 (8), 3090–3095. 10.1021/nl1017173. 20698622

[ref13] Koya A. N.; Li W. Multifunctional charge transfer plasmon resonance sensors. Nanophotonics 2023, 12 (12), 2103–2113. 10.1515/nanoph-2023-0196. PMC11501418 39634047

[ref14] Gerislioglu B.; Ahmadivand A. Functional Charge Transfer Plasmon Metadevices. Research 2020, 2020, 9468692 10.34133/2020/9468692. 32055799 PMC7013279

[ref15] Zhang L.; Zhong X.; Pavlica E.; Li S.; Klekachev A.; Bratina G.; Ebbesen T. W.; Orgiu E.; Samorì P. A nanomesh scaffold for supramolecular nanowire optoelectronic devices. Nat. Nanotechnol. 2016, 11 (10), 900–906. 10.1038/nnano.2016.125. 27454879

[ref16] Prominski A.; Shi J.; Li P.; Yue J.; Lin Y.; Park J.; Tian B.; Rotenberg M. Y. Porosity-based heterojunctions enable leadless optoelectronic modulation of tissues. Nat. Mater. 2022, 21 (6), 647–655. 10.1038/s41563-022-01249-7. 35618824

[ref17] Wei J.; Chen Y.; Li Y.; Li W.; Xie J.; Lee C.; Novoselov K. S.; Qiu C.-W. Geometric filterless photodetectors for mid-infrared spin light. Nat. Photonics 2022, 17 (2), 171–178. 10.1038/s41566-022-01115-7.

[ref18] Priolo F.; Gregorkiewicz T.; Galli M.; Krauss T. F. Silicon nanostructures for photonics and photovoltaics. Nat. Nanotechnol. 2014, 9 (1), 19–32. 10.1038/nnano.2013.271. 24390564

[ref19] Fontana J.; Charipar N.; Flom S. R.; Naciri J.; Piqué A.; Ratna B. R. Rise of the Charge Transfer Plasmon: Programmable Concatenation of Conductively Linked Gold Nanorod Dimers. ACS Photonics 2016, 3 (5), 904–911. 10.1021/acsphotonics.6b00184.

[ref20] Koya A. N.; Lin J. Charge transfer plasmons: Recent theoretical and experimental developments. Applied Physics Reviews 2017, 4 (2), 021104 10.1063/1.4982890.

[ref21] Tobing L. Y. M.; Soehartono A. M.; Mueller A. D.; Czyszanowski T.; Yong K.-T.; Fan W. J.; Zhang D. H. Interplays of Dipole and Charge-Transfer-Plasmon Modes in Capacitively and Conductively Coupled Dimer with High Aspect Ratio Nanogaps. Adv. Opt. Mater. 2021, 9 (22), 2100748 10.1002/adom.202100748.

[ref22] Cheng O. H.-C.; Son D. H.; Sheldon M. Light-induced magnetism in plasmonic gold nanoparticles. Nat. Photonics 2020, 14 (6), 365–368. 10.1038/s41566-020-0603-3.

[ref23] Tao A.; Sinsermsuksakul P.; Yang P. Tunable plasmonic lattices of silver nanocrystals. Nat. Nanotechnol. 2007, 2 (7), 435–440. 10.1038/nnano.2007.189. 18654329

[ref24] Knight M. W.; King N. S.; Liu L.; Everitt H. O.; Nordlander P.; Halas N. J. Aluminum for Plasmonics. ACS Nano 2014, 8 (1), 834–840. 10.1021/nn405495q. 24274662

[ref25] Robatjazi H.; Zhao H.; Swearer D. F.; Hogan N. J.; Zhou L.; Alabastri A.; McClain M. J.; Nordlander P.; Halas N. J. Plasmon-induced selective carbon dioxide conversion on earth-abundant aluminum-cuprous oxide antenna-reactor nanoparticles. Nat. Commun. 2017, 8 (1), 27 10.1038/s41467-017-00055-z. 28638073 PMC5479834

[ref26] Clark B. D.; Jacobson C. R.; Lou M.; Yang J.; Zhou L.; Gottheim S.; DeSantis C. J.; Nordlander P.; Halas N. J. Aluminum Nanorods. Nano Lett. 2018, 18 (2), 1234–1240. 10.1021/acs.nanolett.7b04820. 29272131

[ref27] Clark B. D.; Jacobson C. R.; Lou M.; Renard D.; Wu G.; Bursi L.; Ali A. S.; Swearer D. F.; Tsai A.-L.; Nordlander P.; et al. Aluminum Nanocubes Have Sharp Corners. ACS Nano 2019, 13 (8), 9682–9691. 10.1021/acsnano.9b05277. 31397561

[ref28] Wang C.; Yang W.-C. D.; Raciti D.; Bruma A.; Marx R.; Agrawal A.; Sharma R. Endothermic reaction at room temperature enabled by deep-ultraviolet plasmons. Nat. Mater. 2021, 20 (3), 346–352. 10.1038/s41563-020-00851-x. 33139891 PMC8364736

[ref29] Gérard D.; Gray S. K. Aluminium plasmonics. J. Phys. D: Appl. Phys. 2015, 48 (18), 184001 10.1088/0022-3727/48/18/184001.

[ref30] Yang A.; Hryn A. J.; Bourgeois M. R.; Lee W.-K.; Hu J.; Schatz G. C.; Odom T. W. Programmable and reversible plasmon mode engineering. Proc. Natl. Acad. Sci. U. S. A. 2016, 113 (50), 14201–14206. 10.1073/pnas.1615281113. 27911819 PMC5167184

[ref31] West P. R.; Ishii S.; Naik G. V.; Emani N. K.; Shalaev V. M.; Boltasseva A. Searching for better plasmonic materials. Laser & Photonics Reviews 2010, 4 (6), 795–808. 10.1002/lpor.200900055.

[ref32] Lyaschuk Y.; Indutnyi I.; Myn’ko V.; Romanyuk V.; Mamontova I.; Redko R.; Dusheyko M.; Savchuk Y.; Tochkovyi V.; Shtykalo O.; et al. Aluminum-Based Plasmonic Photodetector for Sensing Applications. Applied Sciences 2024, 14 (11), 4546 10.3390/app14114546.

[ref33] Olson J.; Manjavacas A.; Liu L.; Chang W.-S.; Foerster B.; King N. S.; Knight M. W.; Nordlander P.; Halas N. J.; Link S. Vivid, full-color aluminum plasmonic pixels. Proc. Natl. Acad. Sci. U. S. A. 2014, 111 (40), 14348–14353. 10.1073/pnas.1415970111. 25225385 PMC4210031

[ref34] Gillibert R.; Colas F.; Yasukuni R.; Picardi G.; de la Chapelle M. L. Plasmonic Properties of Aluminum Nanocylinders in the Visible Range. J. Phys. Chem. C 2017, 121 (4), 2402–2409. 10.1021/acs.jpcc.6b11779.

[ref35] Elibol K.; Mangler C.; O’Regan D. D.; Mustonen K.; Eder D.; Meyer J. C.; Kotakoski J.; Hobbs R. G.; Susi T.; Bayer B. C. Single Indium Atoms and Few-Atom Indium Clusters Anchored onto Graphene via Silicon Heteroatoms. ACS Nano 2021, 15 (9), 14373–14383. 10.1021/acsnano.1c03535. 34410707 PMC8482752

[ref36] Gupta T.; Elibol K.; Hummel S.; Stöger-Pollach M.; Mangler C.; Habler G.; Meyer J. C.; Eder D.; Bayer B. C. Resolving few-layer antimonene/graphene heterostructures. npj 2D Mater. Appl. 2021, 5 (1), 53 10.1038/s41699-021-00230-3.

[ref37] Elibol K.; Mangler C.; Gupta T.; Zagler G.; Eder D.; Meyer J. C.; Kotakoski J.; Bayer B. C. Process Pathway Controlled Evolution of Phase and Van-der-Waals Epitaxy in In/In2O3 on Graphene Heterostructures. Adv. Funct. Mater. 2020, 30 (34), 2003300 10.1002/adfm.202003300.

[ref38] Elibol K.; van Aken P. A. Uncovering the Evolution of Low-Energy Plasmons in Nanopatterned Aluminum Plasmonics on Graphene. Nano Lett. 2022, 22 (14), 5825–5831. 10.1021/acs.nanolett.2c01512. 35820031 PMC9335878

[ref39] Elibol K.; van Aken P. A. Hybrid Graphene-Supported Aluminum Plasmonics. ACS Nano 2022, 16 (8), 11931–11943. 10.1021/acsnano.2c01730. 35904978 PMC9413403

[ref40] Barrow S. J.; Rossouw D.; Funston A. M.; Botton G. A.; Mulvaney P. Mapping Bright and Dark Modes in Gold Nanoparticle Chains using Electron Energy Loss Spectroscopy. Nano Lett. 2014, 14 (7), 3799–3808. 10.1021/nl5009053. 24955651

[ref41] Kociak M.; Stéphan O. Mapping plasmons at the nanometer scale in an electron microscope. Chem. Soc. Rev. 2014, 43 (11), 3865–3883. 10.1039/c3cs60478k. 24604161

[ref42] Lourenço-Martins H.; Das P.; Tizei L. H. G.; Weil R.; Kociak M. Self-hybridization within non-Hermitian localized plasmonic systems. Nat. Phys. 2018, 14 (4), 360–364. 10.1038/s41567-017-0023-6.

[ref43] Nicoletti O.; de la Peña F.; Leary R. K.; Holland D. J.; Ducati C.; Midgley P. A. Three-dimensional imaging of localized surface plasmon resonances of metal nanoparticles. Nature 2013, 502 (7469), 80–84. 10.1038/nature12469. 24091976

[ref44] Lingstädt R.; Davoodi F.; Elibol K.; Taleb M.; Kwon H.; Fischer P.; Talebi N.; van Aken P. A. Electron Beam Induced Circularly Polarized Light Emission of Chiral Gold Nanohelices. ACS Nano 2023, 17, 25496 10.1021/acsnano.3c09336. 37992234 PMC10753880

[ref45] Scholl J. A.; Koh A. L.; Dionne J. A. Quantum plasmon resonances of individual metallic nanoparticles. Nature 2012, 483 (7390), 421–427. 10.1038/nature10904. 22437611

[ref46] McClain M. J.; Schlather A. E.; Ringe E.; King N. S.; Liu L.; Manjavacas A.; Knight M. W.; Kumar I.; Whitmire K. H.; Everitt H. O.; et al. Aluminum Nanocrystals. Nano Lett. 2015, 15 (4), 2751–2755. 10.1021/acs.nanolett.5b00614. 25790095

[ref47] Hobbs R. G.; Manfrinato V. R.; Yang Y.; Goodman S. A.; Zhang L.; Stach E. A.; Berggren K. K. High-Energy Surface and Volume Plasmons in Nanopatterned Sub-10 nm Aluminum Nanostructures. Nano Lett. 2016, 16 (7), 4149–4157. 10.1021/acs.nanolett.6b01012. 27295061

[ref48] Eswara Moorthy S. K.; Howe J. M. Temperature dependence of the plasmon energy in liquid and solid phases of pure Al and of an Al-Si alloy using electron energy-loss spectroscopy. J. Appl. Phys. 2011, 110 (4), 043515 10.1063/1.3624735.

[ref49] Palanisamy P.; Howe J. M. Melting and supercooling studies in submicron Al particles using valence electron energy-loss spectroscopy in a transmission electron microscope. J. Appl. Phys. 2011, 110 (2), 024908 10.1063/1.3609063.

[ref50] Palanisamy P.; Sigle W.; Howe J. M. Interfacial plasmon at a singular solid-liquid interface in a partially molten aluminum alloy. Phys. Rev. B 2012, 85 (19), 195305 10.1103/PhysRevB.85.195305.

[ref51] Abe H.; Terauchi M.; Kuzuo R.; Tanaka M. Temperature Dependence of the Volume-Plasmon Energy in Aluminum. Journal of Electron Microscopy 1992, 41 (6), 465–468. 10.1093/oxfordjournals.jmicro.a050994.

[ref52] Zhou M.; Zhang A.; Dai Z.; Feng Y. P.; Zhang C. Strain-Enhanced Stabilization and Catalytic Activity of Metal Nanoclusters on Graphene. J. Phys. Chem. C 2010, 114 (39), 16541–16546. 10.1021/jp105368j.

[ref53] Förster G. D.; Rabilloud F.; Calvo F. Atomistic modeling of epitaxial graphene on Ru(0001) and deposited ruthenium nanoparticles. Phys. Rev. B 2015, 92 (16), 165425 10.1103/PhysRevB.92.165425.

[ref54] Ehrenreich H.; Philipp H. R.; Segall B. Optical Properties of Aluminum. Phys. Rev. 1963, 132 (5), 1918–1928. 10.1103/PhysRev.132.1918.

[ref55] Duan H.; Fernández-Domínguez A. I.; Bosman M.; Maier S. A.; Yang J. K. W. Nanoplasmonics: Classical down to the Nanometer Scale. Nano Lett. 2012, 12 (3), 1683–1689. 10.1021/nl3001309. 22313285

[ref56] Zengin G.; Wersäll M.; Nilsson S.; Antosiewicz T. J.; Käll M.; Shegai T. Realizing Strong Light-Matter Interactions between Single-Nanoparticle Plasmons and Molecular Excitons at Ambient Conditions. Phys. Rev. Lett. 2015, 114 (15), 157401 10.1103/PhysRevLett.114.157401. 25933338

[ref57] Bosman M.; Watanabe M.; Alexander D. T. L.; Keast V. J. Mapping chemical and bonding information using multivariate analysis of electron energy-loss spectrum images. Ultramicroscopy 2006, 106 (11), 1024–1032. 10.1016/j.ultramic.2006.04.016. 16876322

[ref58] Hohenester U. Simulating electron energy loss spectroscopy with the MNPBEM toolbox. Comput. Phys. Commun. 2014, 185 (3), 1177–1187. 10.1016/j.cpc.2013.12.010.

[ref59] Dwivedi R.; Aradian A.; Ponsinet V.; Vynck K.; Baron A. Effective-medium description of dense clusters of plasmonic nanoparticles with spatial dispersion. Phys. Rev. A 2024, 109 (2), 023507 10.1103/PhysRevA.109.023507.

[ref60] Schmidt F. P.; Ditlbacher H.; Hofer F.; Krenn J. R.; Hohenester U. Morphing a Plasmonic Nanodisk into a Nanotriangle. Nano Lett. 2014, 14 (8), 4810–4815. 10.1021/nl502027r. 25000389 PMC4133183

[ref61] Jia-Sheng W.; Lancaster M. J. Microstrip Filters for RF/Microwave Applications [Book Review]. IEEE Microwave Magazine 2002, 3 (3), 62–65. 10.1109/MMW.2002.1028365.

[ref62] Haas J.; Ulrich F.; Hofer C.; Wang X.; Braun K.; Meyer J. C. Aligned Stacking of Nanopatterned 2D Materials for High-Resolution 3D Device Fabrication. ACS Nano 2022, 16 (2), 1836–1846. 10.1021/acsnano.1c09122. 35104934

[ref63] Bitton O.; Gupta S. N.; Houben L.; Kvapil M.; Křápek V.; Šikola T.; Haran G. Vacuum Rabi splitting of a dark plasmonic cavity mode revealed by fast electrons. Nat. Commun. 2020, 11 (1), 487 10.1038/s41467-020-14364-3. 31980624 PMC6981195

