# Supplementary material for: Unlocking Unexpected Charge Transfer Pathways in Interconnected Nanostructures
Source: ACS Appl Mater Interfaces. 2024 Oct 15;16(42):57501–11. doi: 10.1021/acsami.4c12205 (PMC11503614; doi:10.1021/acsami.4c12205)
Supplement: Supplementary file 1 — am4c12205_si_001.pdf [file am4c12205_si_001.pdf]

SUPPORTING INFORMATION

Unlocking unexpected charge transfer pathways in interconnected nanostructures

Kenan Elibol, Marko Burghard, Tobias Heil and Peter A. van Aken*

Max Planck Institute for Solid State Research, Heisenbergstr. 1, 70569 Stuttgart, Germany.

*Corresponding author: k.elibol@fkf.mpg.de

Note S1. Substrate effects

The optical characteristics of metal NPs are profoundly influenced by the substrate upon which they are immobilized, as extensively documented in literature.[1] The selection of a substrate, whether dielectric or metal, introduces a defining context that distinctly shapes the plasmon resonances of the NPs.[1, 2] Parameters such as the substrate's dielectric constant and refractive index of the substrate wield considerable influence, modifying the resonance frequency and intensity of plasmon modes.[3] This interaction often culminates in the creation of hybrid plasmonic structures, where the plasmon modes of metal NPs intricately couple with those of the substrate, yielding novel and tunable optical properties.[4]

While EELS stands out as an excellent method for unraveling the plasmon resonances of complex NPs, the underlying membrane can introduce challenges due to mode mixing, complicating the identification of plasmon modes.[4, 5] However, this concern is effectively mitigated through the use of an ultrathin membrane material such as single-layer graphene.[4, 6, 7] Beyond serving as an ideal template for metal cluster synthesis, ,[8, 9] graphene proves to be an outstanding substrate for

plasmonic studies, inducing a minimal redshift of 0.09 eV.[6, 7, 10] Illustrated in Figure S1a-c, our integration of Al nanocrosses on an atomically clean suspended monolayer graphene membrane demonstrates the efficacy of this approach. Notably, thermal annealing at 300 ˚C in air for 15 min yields extensive clean areas on the graphene substrate, despite the persistence of residual hydrocarbon contamination. Even in the presence of accumulated charges on the graphene surface, the material remains charge-neutral at the charge neutrality point unless biased,[6] ensuring minimal alteration of CTP resonances in the underlying nanostructures.

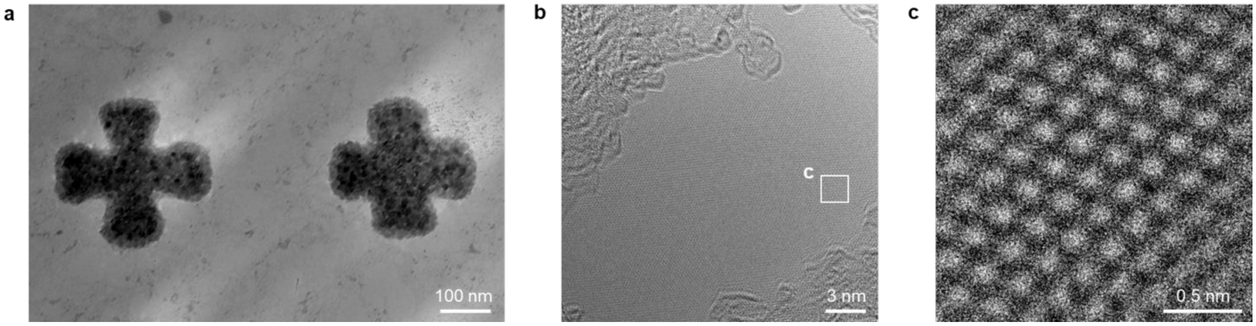

Figure S1. Close-up TEM images of Al nanocrosses and graphene underlying. (a) TEM image of the nanocrosses with different junction areas. (b,c) TEM images of the graphene supporting Al nanocrosses.

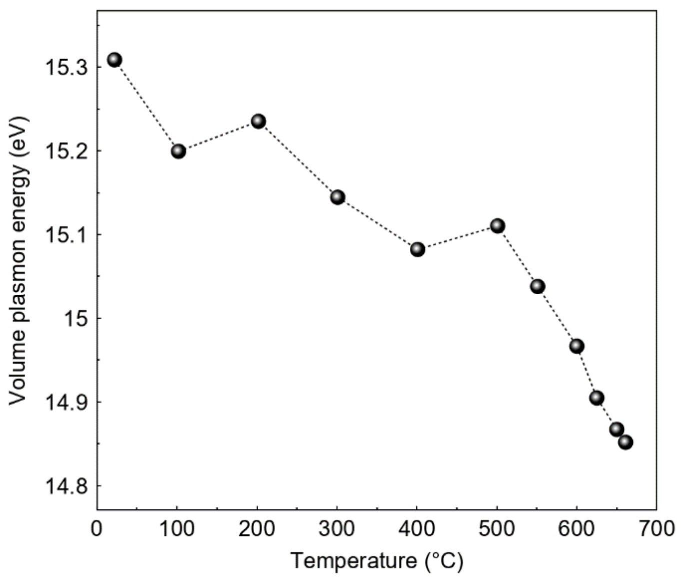

Figure S2. Calculated volume plasmon energy as a function of temperature. The thermal expansion coefficient of Al was obtained from P. Palanisamy et. al.[11]

Note S2. Details of LC circuit model

The LC circuit model stands as an indispensable and insightful theoretical construct for decoding the intricacies of plasmon resonances in metal NPs.[12-14] This model conceptualizes the metallic NP as a miniature LC (inductor-capacitor) circuit, where the inductance captures to the kinetic energy stemming from the motion of free electrons, and the capacitance arises from the electrostatic energy resulting from charge separation within the NP. The resonant frequency of the LC circuit, a key parameter, is intricately tied to the NP's size, shape, and the dielectric environment it inhabits.
This theoretical framework empowers us to predict and analyze the plasmon resonances of metal NPs, thereby affording a profound understanding of their optical properties.[13] The inherent simplicity and efficacy of the LC circuit model render it a powerful tool for unraveling the fundamental principles that underlie plasmon resonances in metal NPs.[15]

As previously discussed, Al nanocrosses with uniform interconnects comport themselves akin to a coupled LC circuit.[6, 15] Figure S3 offers an LC circuit model corresponding to the nanocross depicted in Figure 1e of the manuscript. The schematic reveals that the junction area in Al nanocrosses, represented by the blue region, can be predicted from the geometry of the nanostructures. The observed blue-shift in CTP resonance with increasing junction area is attributed to the augmented kinetic energy, a consequence of more electrons contributing to the electrical conduction in the junction. Moreover, the lifetime of the CTP exhibits a monotonic decrease with expanding junction area. Remarkably, this coupled LC circuit approach aligns with both experimental findings and simulations.

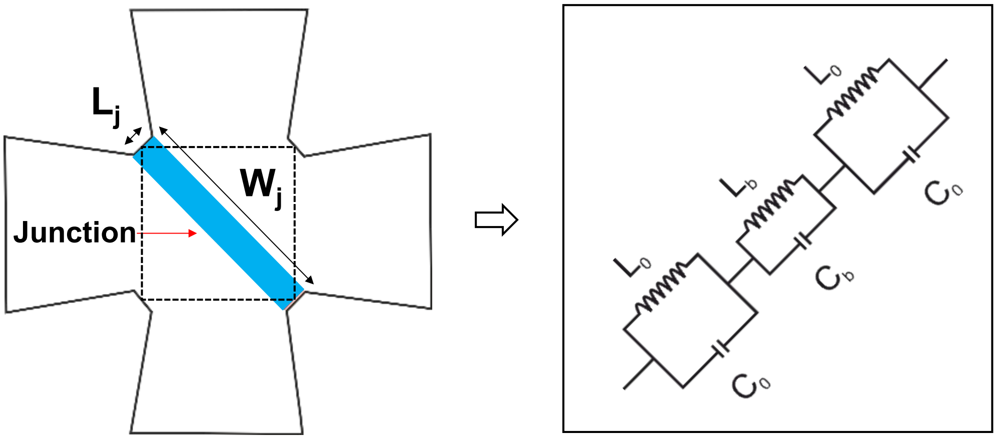

Figure S3. Equivalent LC circuit model for an Al nanocrosses with a uniform junction. Schematic corresponding to the LC circuit model of a nanocross with a uniform junction. Here, L_j and W_j represent the length and width of the junction. The dashed black frame represents the interconnect area.

Note S3. LSPRs

Figure S4a,b shows an HAADF image capturing the uniform junction of the nanocross and its corresponding model used in BEM simulations. The experimental and simulated EEL spectra, acquired from both the corner and the center of Al nanocrosses, reveals the emergence of different plasmon resonances (blue and black triangle-marked peaks) distinct from the CTP resonance marked by a red triangle (Figure S4c).

Figure S4d,e, shows EELS maps, conclusively identifying these modes as LSPRs within Al nanocrosses. To discern these LSPR modes accurately, we turn to computed eigenmodes corresponding to the features presented in Figure S4e. The peak denoted by a blue triangle in EEL spectra aligns with a 3λ/2 resonance, evidenced by the observed dipole formation in each nanoprism and the resultant net dipole moment in the nanocross (Figure S4f). Conversely, the mode exited at the center of the nanocross(Figure S4a-c) manifests as an λ resonance, characterized by a zero net dipole moment (Figure S4f).

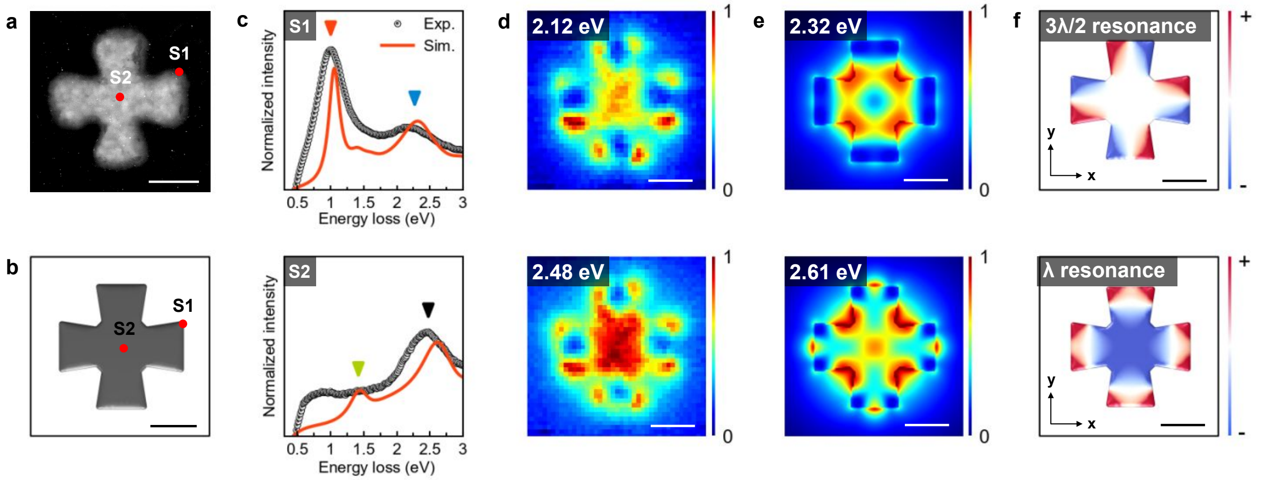

Figure S4. LSPRs of the Al nanocross shown in Figure 1e in the manuscript. (a,b) HAADF image and its corresponding model used in BEM simulations, respectively. (c) Experimental and

simulated EEL spectra obtained at the positions marked with red dots on panels (a) and (b). The red, blue, black and green triangles mark the CTP mode, 3λ/2 resonance, λ resonance and IBT, respectively. (d,e) Experimental and simulated EELS maps obtained at indicated energies, respectively. (f) Simulated eigenmodes corresponding to the plasmon resonances shown in e. The scale bars are 100 nm (a,b,d,e,f).

Note S4. Impact of junction area on CTPs and LSPRs of Al nanocrosses

Figure S5a,b provides a comprehensive view of both experimental and simulated EEL spectra of Al nanocrosses, showcasing the impact of varying interconnect areas on the plasmonic response. The dimensions of our computational models in BEM simulations closely mirror those extracted from the corresponding experimental structures. In the experimental setup, the electron beam is positioned at the right edge of the nanoprisms to capture EEL spectra from nanocrosses featuring diverse interconnect areas, as illustrated in the insets in Figure S5a,b.

The alignment between experimental and simulated EEL spectra unveils a conspicuous redshift in the CTP resonance of nanocrosses, mirroring the augmented interconnect areas. This redshift, consistent with our LC circuit model, finds its explanation in the enhanced kinetic energy stored within larger junctions. The increase in the number of electrons within these expanded interconnects contributes to the observed blueshift in CTP resonances, as elucidated in our previous discussion.

Intriguingly, while the resonance energy of the bonding dipolar LSPR remains unaffected by interconnect area variations (marked with a blue triangle in Figure S5c), antibonding dipole LSPRs exhibit a subtle blueshift with the expansion of nanocross junction areas (marked with a black triangle in Figure S5c). This nuanced response further underscores the exquisite control and tunability achieved in Al nanocrosses, laying the groundwork for tailoring their plasmonic properties for diverse applications.

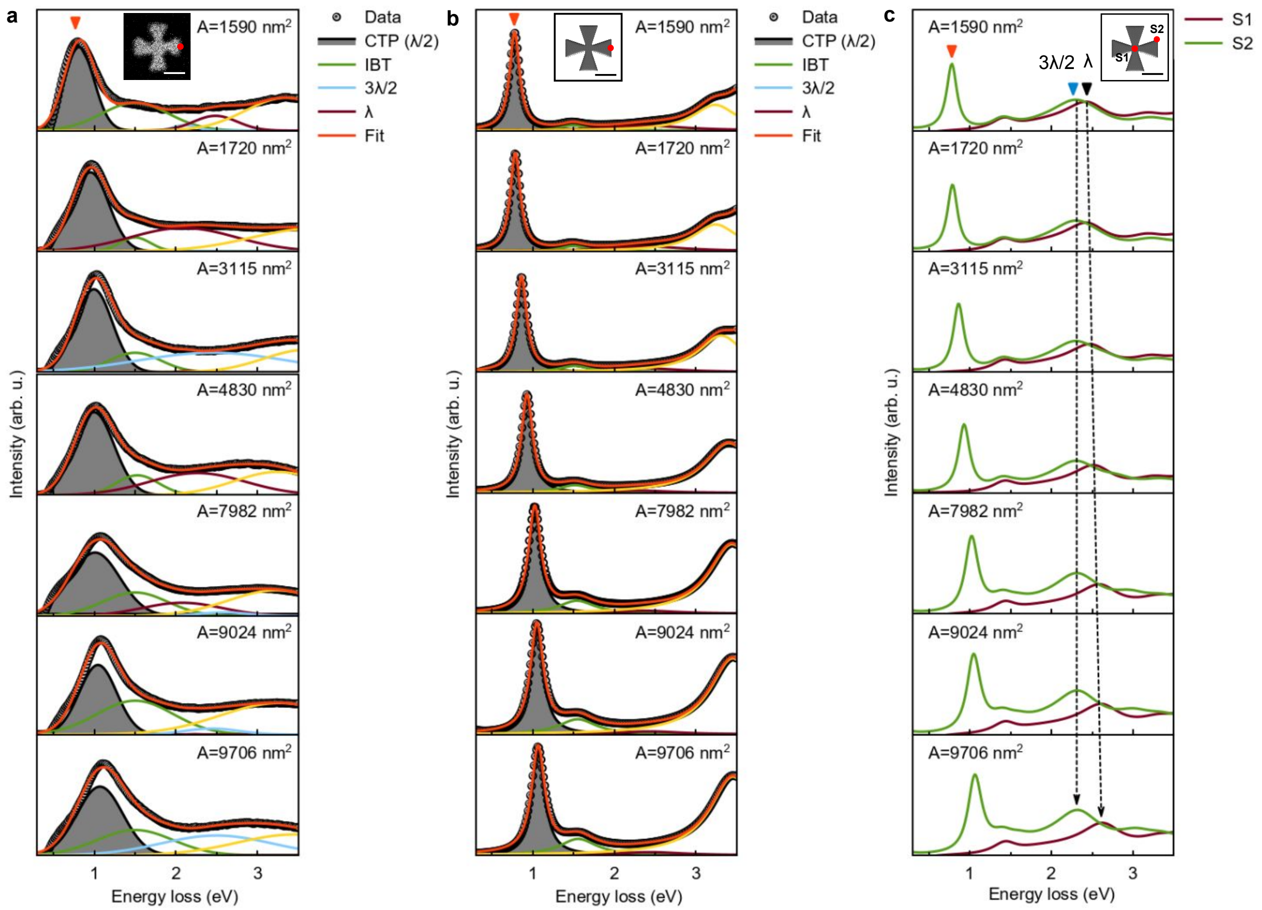

Figure S5. EEL spectra of Al nanocrosses with different interconnect areas. (a) EEL spectra acquired on nanocrosses with different interconnect areas. The spectra are obtained from the edge of the nanoprism on the right side of the nanocross (marked with a red spot on the HAADF image shown in the the inset). The peak positions and linewidths in (a) were determined by fitting a Gaussian to the spectra. (b,c) Simulated EEL spectra derived from the edge, tip and center of nanocrosses with different interconnect areas (marked with red spots on the models shown in the inset), respectively. The peak positions and linewidths in (b) were determined by fitting a Lorentzian to the spectra. The scale bars are 100 nm (a,b,c).

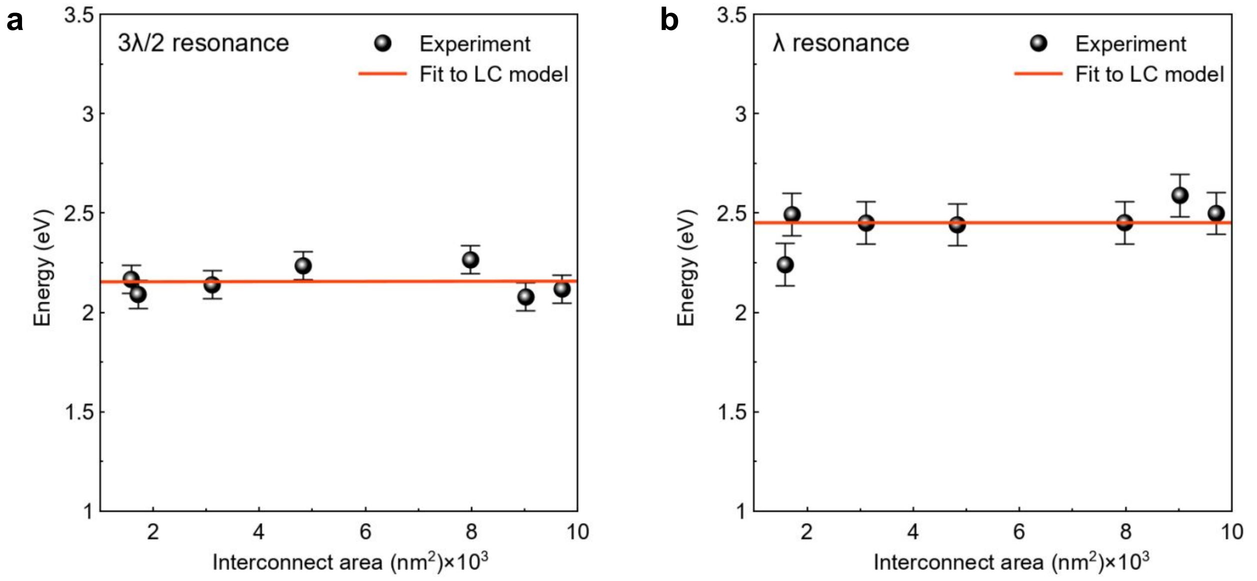

Figure S6. Energies of (a) 3λ/2 and (b) λ resonances as a function of interconnect area. Solid red lines represent fitting to the LC circuit model.

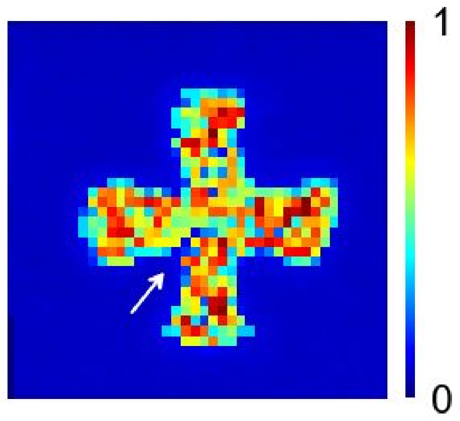

Figure S7. Volume plasmon map acquired at 14.4 eV.

Note S5. Effect of nano-trench on λ resonance and VP

In Figure S8a, we present a comprehensive EEL spectrum analysis encompassing an extensive energy range of 0.3-17 eV for an Al nanocross featuring an electron-beam-induced nano-trench. Our analysis focuses on spectra acquired from both the left and right edges of the nanocross (see inset in Figure S8a), revealing pronounced peaks corresponding to CTP and VP resonances, highlighted by red and purple triangles, respectively. A notable observation unfolds as we examine the VP resonance, remaining steadfast in its position across both spectra. Intriguingly, a discernible redshift manifests in the CTP resonance observed within the spectra acquired proximal to the nano-trench. Figure S8b further supplements our findings, indicating that the resonance energy of λ resonance maintains constancy, unaffected by the introduction of a nano-trench within the Al nanocrosses.

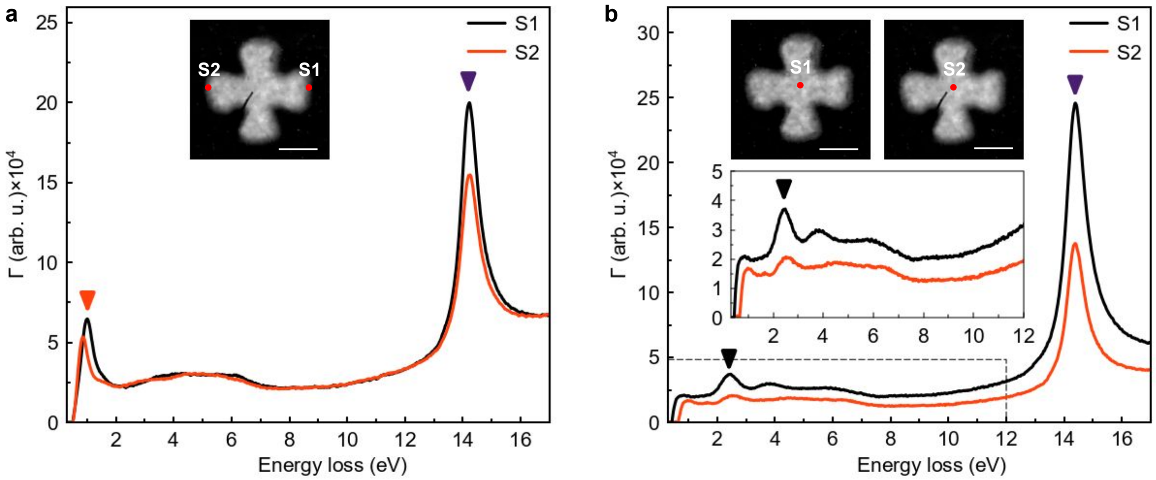

Figure S8. Antibonding dipole and VP modes after creation of a nano-trench. (a) EEL spectra acquired at the positions marked with red dots. The red and purple triangles point to the CTP and VP resonances, respectively. (b) EEL spectra recorded at the position marked with a red dot on an Al nanocross before and after trench creation. The black and purple triangles point to the λ resonance and VP. The inset shows the close-up of the area shown in the dashed frame. The scale bars are 100 nm (a,b).

Note S6. CTPs in Al nanocrosses with an e-beam-induced nano-trench

The EEL spectra obtained from both edges of an Al nanocross with a uniform junction are plotted in Figure S9a,b. Both experimental and simulated EEL spectra are deconvolved using Gaussian or Lorentzian curve fitting. Since the spectra obtained from both the left and right edges of nanocross are identical (Figure 2a), we show the spectra obtained from the right edge. Figure S9a,b shows that a single CTP resonance is excited when the junctions in Al nanocrosses are uniform. The linewidths of the experimental and simulated CTP resonances for the nanocross with a uniform junction are found to be 0.38 and 0.20 eV from the fit.

Upon formation of an e-beam-induced nano-trench within the interconnect of the same IAlN, we observe the formation of two CTP modes (CTP-L and CTP-H) resonating at slightly different energies (Figure S11c,d). As observed in Al nanocrosses with oxidation-induced nano-trench, the CTP-L resonance is excited at the edge close to the nano-trench while the CTP-H with higher resonance energy is probed on the opposite edge far from the nano-trench in Al nanocrosses with e-beam-induced nano-trench. The experimental linewidths of the CTP-L and CTP-H resonances are measured to be 0.3 and 0.45 eV. The simulated linewidths of CTP-L and CTP-H resonances are 0.14 and 0.22 eV. The discrepancy is again due to the imperfections of the experimental structure. Both experimental and simulated data indicate that the lifetime of the CTP mode in the nanocross with a uniform junction is higher than the CTP-H mode excited after the formation of a nano-trench. However, the CTP-L lifetime is much higher than the CTP-H lifetime.

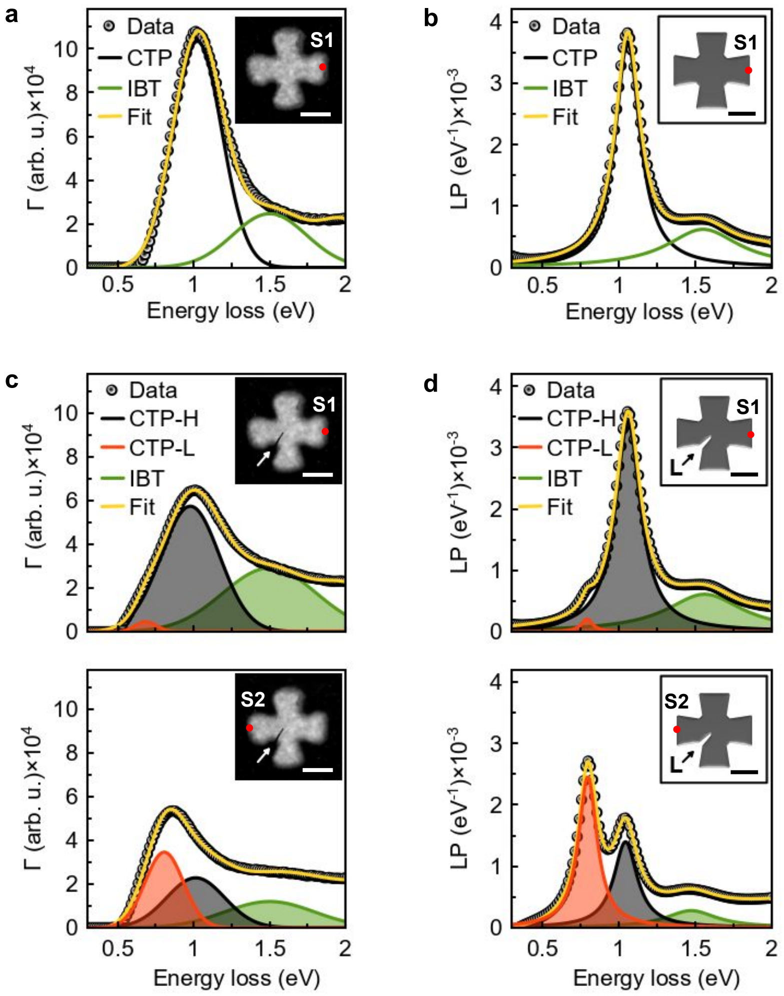

Figure S9. Fitting for EEL spectra of an Al nanocross without and with a deformed junction. (a,b) Fitting to experimental and simulated EEL spectra obtained on an Al nanocross without deformation in its junction area. (c,d) Fitting to experimental and simulated EEL spectra obtained on an Al nanocross with an e-beam-induced trench created in its junction area. A Gaussian fit is applied to the EEL spectra to define the peak positions in the experimental data while a Lorentzian fit is used to extract the peak positions in the simulated EEL spectra. The experimental and simulated linewidths of the nanocross with a uniform junction shown in (a) are 0.38 eV and 0.20 eV, respectively. The experimental linewidths of the CTP-L and CTP-H resonances in (c) are 0.30 eV and 0.45 eV, respectively. The simulated linewidths of CTP-L and CTP-H resonances in (d) are 0.14 eV and 0.22 eV, respectively. The scale bars are 100 nm (a,b,c,d).

Note S7. Formation of two oscillators via junction distortion

When an asymmetry is introduced within the junction area, as depicted in Figure S10, the CTP mode excited near the distorted region exhibits a discernible alteration in comparison to the initial CTP mode. Meanwhile, the CTP mode excited at the opposite edge close to the undistorted area demonstrates minimal change. This observation implies that the system enables the excitation of two CTP modes at different energies, suggesting a behavior analogous to that of two coupled oscillators. In the simplified model shown in Figure S10, the junction distortion induced by nano-trench is shown in the yellow colored area.

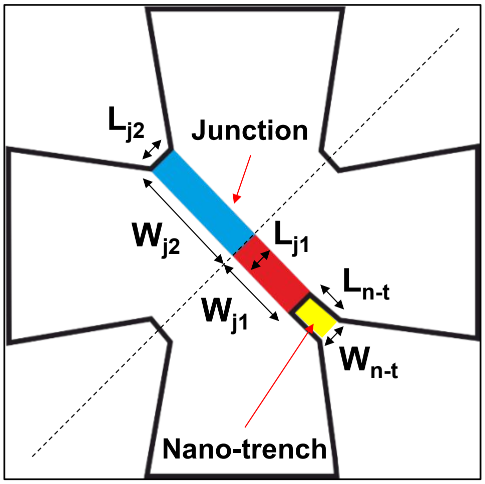

Figure S10. Formation of two oscillators via junction distortion. A schematic showing a single trenched Al nanocross acting as two coupled bowties. The junction lengths (L_{j1} and L_{j2}) are same whereas there is a difference between junction widths (W_{j1} and W_{j2}). The L_{n-t} and W_{n-t} are the length and width of the nano-trench.

Note S8. CTP modes in Al nanocrosses deformed by focused e-beam irradiation

To validate the emergence of CTPs resonating at different energies in Al nanocrosses with distorted junctions, we induce e-beam-induced nanotrenches in another Al nanocross sample with a larger junction area, as shown in Figure S11a-d. Consistent with the results presented in Figure 4, we observe the excitation of both CTP-L and CTP-H upon the generation of a nanotrench within the junction area by focused e-beam irradiation, as shown in Figure S11e-i.

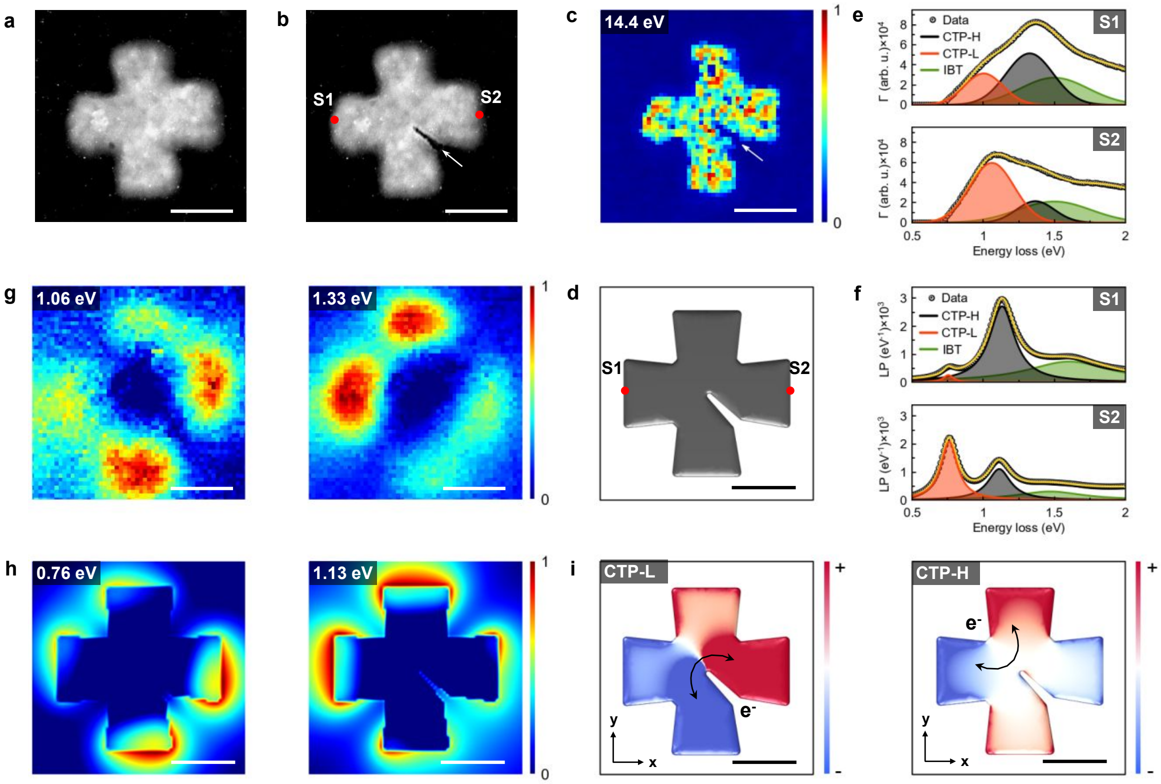

Figure S11. CTP modes in Al nanocrosses deformed by focused e-beam irradiation. (a,b) HAADF image of an Al nanocross before and after the creation of an e-beam-induced trench in its junction. The junction area and trench length in the experimental structure are 9216 nm^2 and 58 nm, respectively. (c) VP map for the Al nanocross shown in (b). The white arrows on the VP map point to the trench created by the electron beam. (d) Model corresponding to the experimental structure

in (b). (e,f) Experimental and simulated EEL spectra recorded on different positions marked with red dots in panels (b) and (d). A Gaussian fit is applied to the EEL spectra to define the peak positions in the experimental data, while a Lorentzian fit is used to extract the peak positions in the simulated EEL spectra. The experimental linewidths of the CTP-L and CTP-H resonances in (e) are 0.37 eV and 0.39 eV, respectively. The simulated linewidths of the CTP-L and CTP-H resonances in (f) are 0.14 eV and 0.22 eV, respectively. (g,h) Experimental and simulated EELS maps obtained at different energies. (i) Simulated eigenmodes corresponding to the plasmon resonances shown in (h). The scale bars are 100 nm (a-d, g-i).

Note S9. CTP modulation through interconnect oxidation

We further inspect Al nanocrosses with partially oxidized junctions using low-loss EELS measurements to better understand the effect of junction distortion on CTP resonances. Al nanocrosses are patterned with different electron doses to adjust the dimensions of their junctions. Our results show that the formation of junctions between nanoprisms within the nanocrosses begins when the beam dose exceeds 1440 μC/cm² (Figure S12). Beyond doses of 1488 μC/cm², the majority of nanoprisms are observed to merge within the nanocrosses. Notably, at relatively low beam doses of 1440 μC/cm², some nanoprisms remain closely spaced while others merge within the nanocrosses (Figure S12), which is attributed to the proximity effect during electron beam lithography. Upon exposure to the ambient environment, a native oxide layer forms on the Al surface.[16-18] The oxide layer formed between the metallic Al and the surrounding medium acts as a protective barrier and prevents any further oxidation of the Al.[7, 18] In addition to the native oxide, we identify fully oxidized areas within the junctions of Al nanocrosses patterned at beam doses ranging from 1440 to 1632 μC/cm² (Figure S13). The thickness of the native oxide layer is estimated to be ~10 nm in our samples (Figure S13c). However, we find that the thickness of the fully oxidized region within the junction is much higher than that of the native oxide (Figure S13d,e). This is due to the volume expansion during oxidation,[8] which is further supported by ambient thermal annealing at 300 °C.

We propose that the fully oxidized region observed within the junction results from the merging of oxide layers formed on the sidewalls of closely spaced nanoprims. The merging of nanoprims is further supported by the volume expansion of the oxide layer between the closely spaced nanoprims (Figure S13f). As shown in Figure S12b, closely spaced nanoprisms are obtained when Al nanocrosses are patterned at beam doses ranging from 1440 to 1632 μC/cm². Consequently, a small

volume within the junction appears fully oxidized for Al nanocrosses patterned at intermediate radiation doses of 1440 to 1632 μC/cm² (Figure S13a-e). The observed density of Al nanocrosses with oxidized junctions is found to be 14% (out of a total of 50 observations).

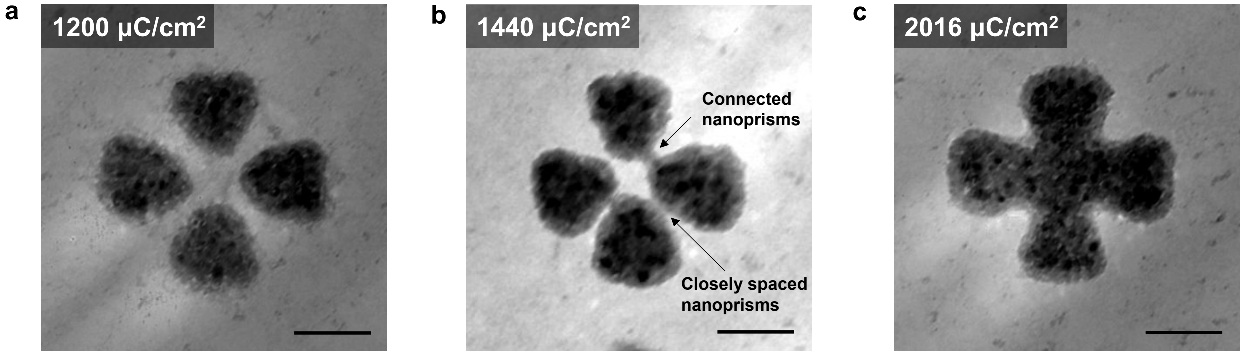

Figure S12. TEM images of Al nanocrosses patterned at different beam doses. (a,b,c) TEM images for Al nanocrosses patterned at the beam doses of 1200, 1440 and 2016 μC/cm^2 respectively. The scale bars are 100 nm (a,b,c).

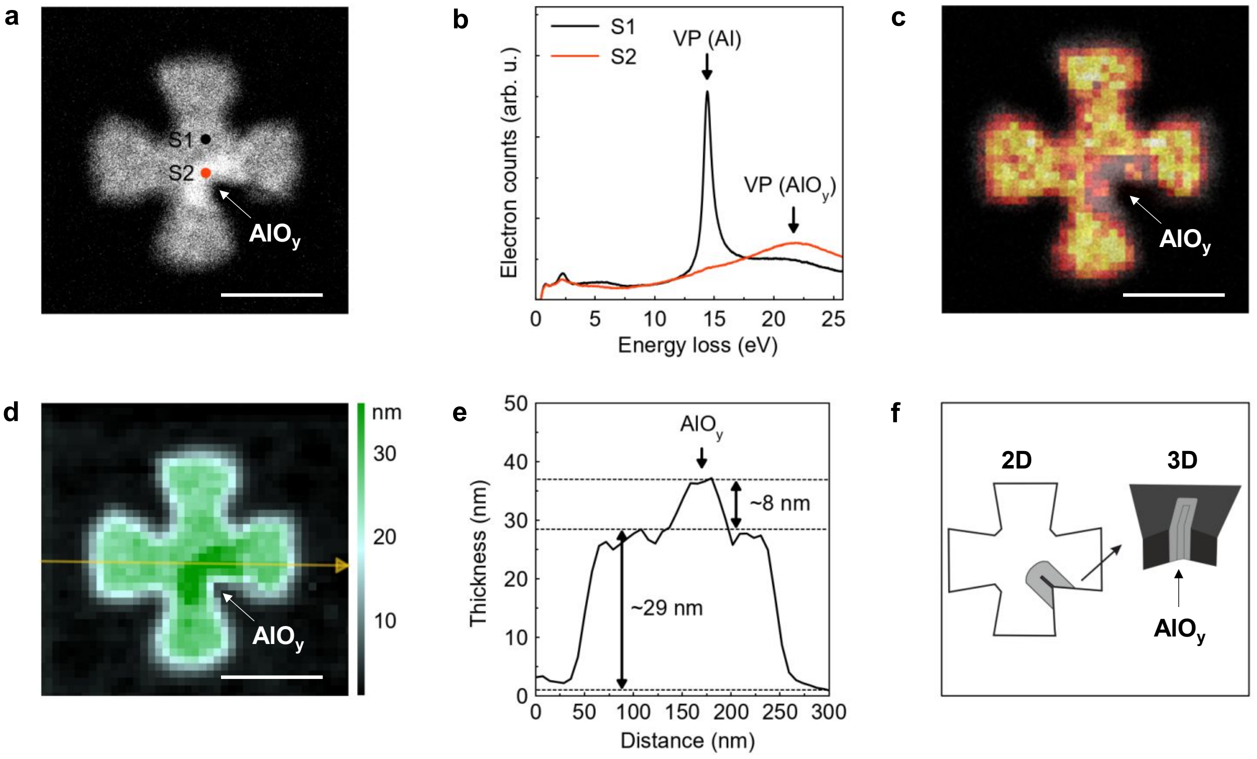

Figure S13. Formation of AlO_y within Al nanocrosses. (a) HAADF image of the Al nanocross with a partially oxidized junction. The Al nanocross is patterned at the beam dose of 1536 $\mu C/cm^2$. (b) EEL spectra acquired from the spots marked by black and red dots in (a). (c) Superimposed HAADF image and VP map. (d) Thickness map for the Al nanocross in (a). (e) Line profile obtained along the semitransparent yellow line on the thickness map. (f) A simplified model showing the partially oxidized junction depicted in both 2D and 3D perspectives. The scale bars are 100 nm (a,c,d).

The HAADF image in Figure S14a captures the partially bright appearance of the Al nanocross junction, indicative of the oxidation. Similar to the nano-trench effect, the oxidation of the junction within the Al nanocross also results in the emergence of a new, lower energy CTP mode (Figure S14a). The spatially resolved VP map shows that the metallic region within the junction is partially destroyed by the oxidation process (Figure S14b). To confirm the presence of an oxide layer, EDS

spectra are acquired on the Al nanocross (Figure S14c). EDS maps generated at the specific energies corresponding to elemental Al and oxygen (O) confirm the partial oxidation of the junction (Figure S14d,e). In this context, the oxidation induces separation between adjacent nanoprisms in Al nanocrosses.

The presence of this new CTP-L mode is confirmed in simulated EEL spectra derived from a structural model (Figure S14f). Since oxidation-induced distortion causes separation between adjacent nanoprisms within the nanocrosses, we choose to use models with nanocrosses with nano-trenches to simplify the simulations. Excitation at the edge far from the nano-trench leads to a pronounced excitation of CTP-H, while CTP-L is formed with weaker intensity. Conversely, excitation near the nano-trench edge favors CTP-L, revealing a dynamic interplay in charge transfer pathways within the Al nanocrosses. As observed in Al nanocrosses with nano-trecnhes, EELS maps extracted at ω_{CTP-L} and ω_{CTP-H} show that the electron transfer occurs predominantly between two adjacent nanoprisms (Figure S14g,h,i). Using linewidths obtained from curve fitting, the lifetimes of CTP-L (τ_{CTP-L}) and CTP-H (τ_{CTP-H}) excited at the oxidized Al nanocross are determined to be 2.01 fs and 1.44 fs, respectively (Figure S15).

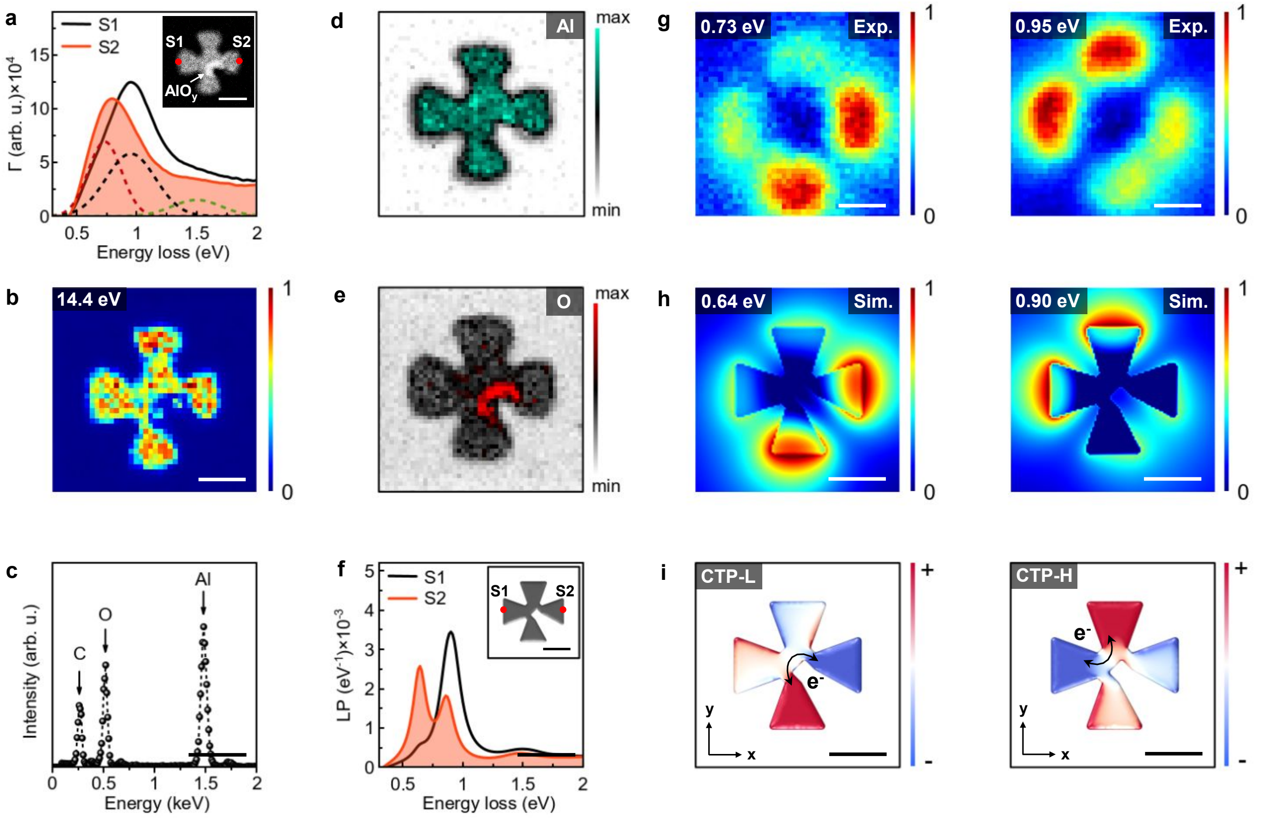

Figure S14. Tailoring of CTPs and charge transfer pathways by interconnect oxidation. (a) EEL spectra obtained from positions marked by red dots on the HAADF image of an Al nanocross. The dashed red, black and green lines represent Gaussian fitting for the spectra obtained from position S2. (b) VP map corresponding to the Al nanocross shown in (a). (c) EDS spectra recorded for the nanocross in (a). (d,e) EDS maps illustrating the elemental Al and O distribution on the nanocross in (a). (f) Simulated EEL spectra derived at locations marked by red dots on the model corresponding to the Al nanocross in (a). (g,h) Spatially resolved experimental and simulated EELS maps extracted at energies identified from the EEL spectra in (a) and (f). (i) Calculated eigenmodes corresponding to the modes observed in simulated EELS maps in (h). Scale bars are 100 nm (a-c,e-i).

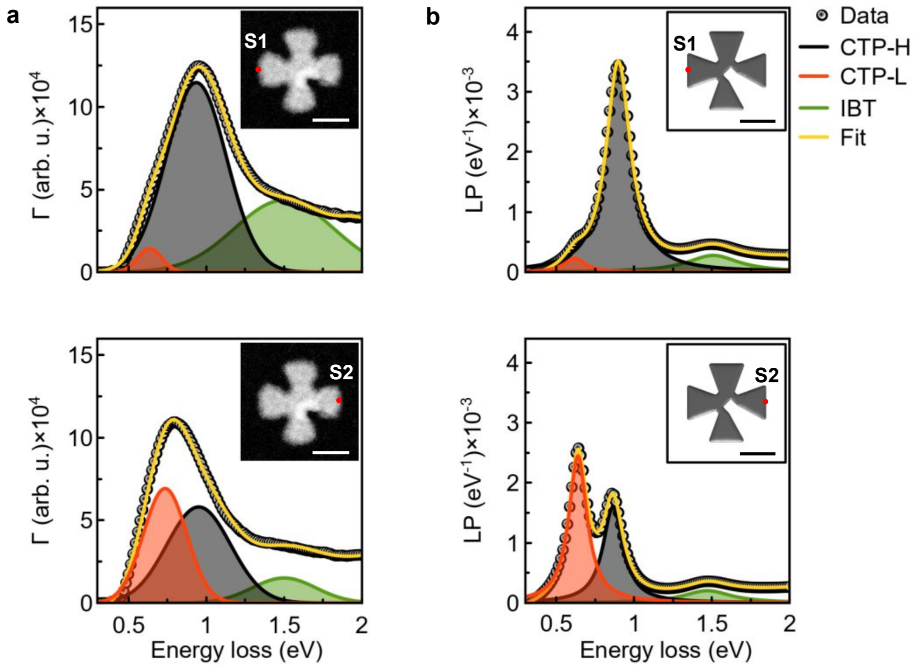

Figure S15. Fitting of EEL spectra from an Al nanocross with a deformed junction. (a,b) Experimental and simulated EEL spectra obtained on different positions marked with red dots on the HAADF images and models, respectively. Gaussian fitting is applied to EEL spectra to define the peak positions in the experimental data, while a Lorentzian fitting is used to extract the peaks positions in the simulated EEL spectra. The experimental linewidths of CTP-L and CTP-H resonances in (a) are 0.33 eV and 0.46 eV, respectively. The simulated linewidths of CTP-L and CTP-H resonances in (b) are 0.14 eV and 0.19 eV, respectively. The scale bars are 100 nm (a,b).

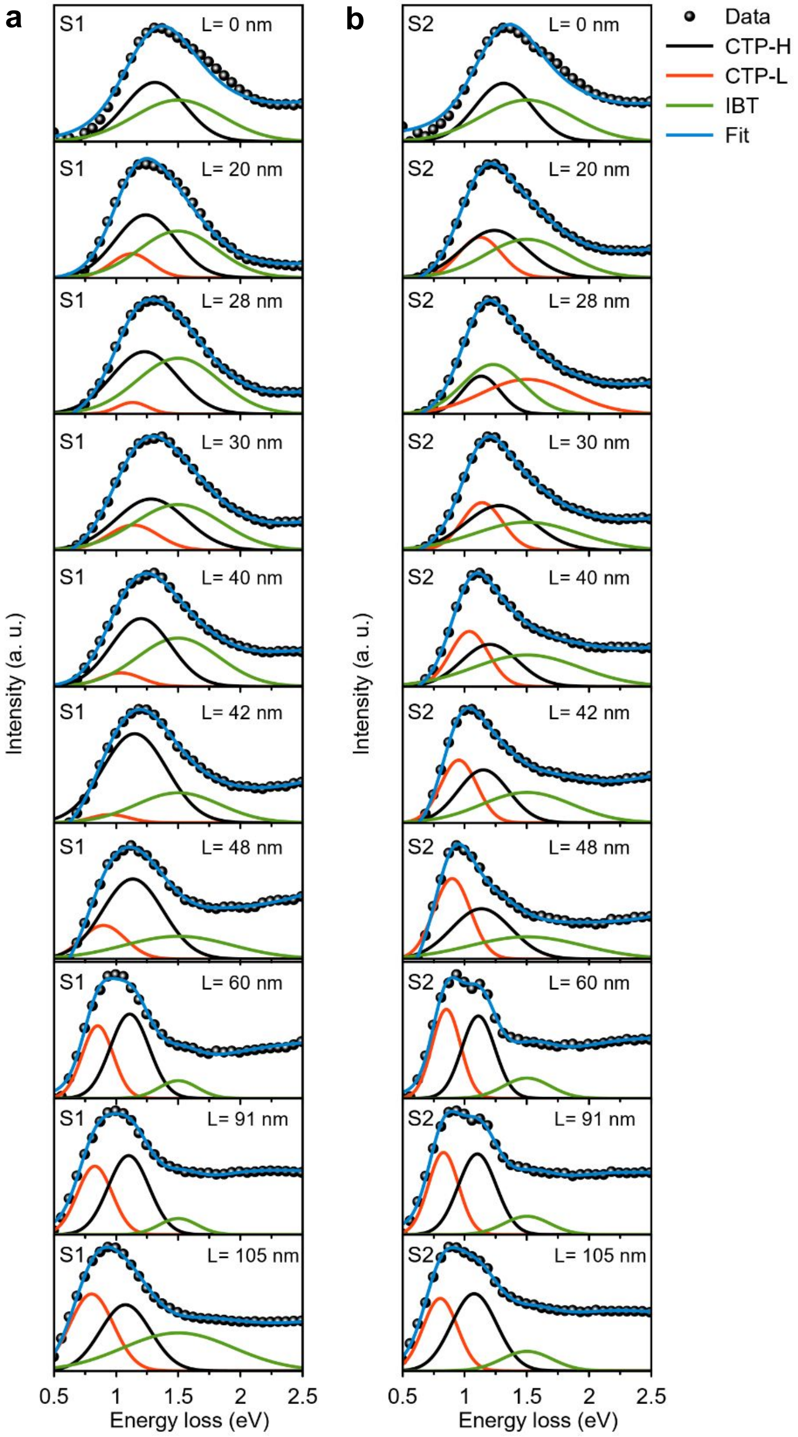

Figure S16. EEL spectra deconvolved by Gaussian fitting. Spectra are acquired from the marked spots (a) S1 and (b) S2 on the Al nanocross with different trench lengths as shown in Figure 3a.

Note S10. Formation of DGM resonance

Figure S17a-e shows EELS simulations for Al nanocrosses including two nano-trenches. The lengths of both trenches are varied from 0 to 64 nm. Compared to Al nanocrosses with a single trench (Figure 4a), the EEL spectra derived from both the left and right edges of Al nanocrosses with double trench are identical (Figure S17b). With increasing trench length, CTP-L red-shifts, while CTP-H remains almost stationary. As observed in Figure 4a, we notice the formation of a new mode, marked by a blue triangle in Figure S17b**,** when both trench lengths are larger than 24 nm. This new plasmonic mode is identified as a low-energy dipolar gap mode (DGM), which is a bonding type, from the EELS maps and plasmonic eigenmodes in Figure S17c,d. Similar to Al nanocrosses with a single trench (Figure 4d), the CTP-H resonances do not vary with the change in trench length but the CTP-L resonance decays nonlinearly with the increase in trench length (Figure S17e). Interestingly, we observe an exponential decrease of the DGM resonance with increasing trench length (Figure S17e). To better assess the plasmon resonances of Al nanocrosses, we also investigate the plasmon modes of nanocrosses without a connection between two coupled nanoprisms (Figure S17f). The gap size between the connected nanoprims is varied from 4 to 49 nm. For the gap sizes smaller than 49 nm, a new mode marked with a blue triangle appears close to the CTP mode marked with a black triangle (Figure S17g). Similar to the Al nanocross with a double trench, we observe a DGM mode at the resonance energies slightly lower than the CTP-H resonance (Figure S17h,i). We find that a CTP-L resonance does not form when there is no junction between the connected nanoprisms. For the gap sizes larger than 49 nm, the DGM has no significant impact on CTP. The resonance energy of CTP-H increases slightly, while the energy of DGM decreases exponentially with increasing gap size (Figure S17j). In contrast to Al nanocrosses with a single trench, the DGM mode can appear in nanocrosses with double trenches when their trench lengths are larger than 24 nm. In addition, the excitation of DGM leads to a nonlinear decay of

CTP-L in the double-trenched Al nanocross (Figure S17e), whereas the CTP-L resonances in the single-trenched Al nanocross are monotonically reduced with increasing trench length (Figure 4d).

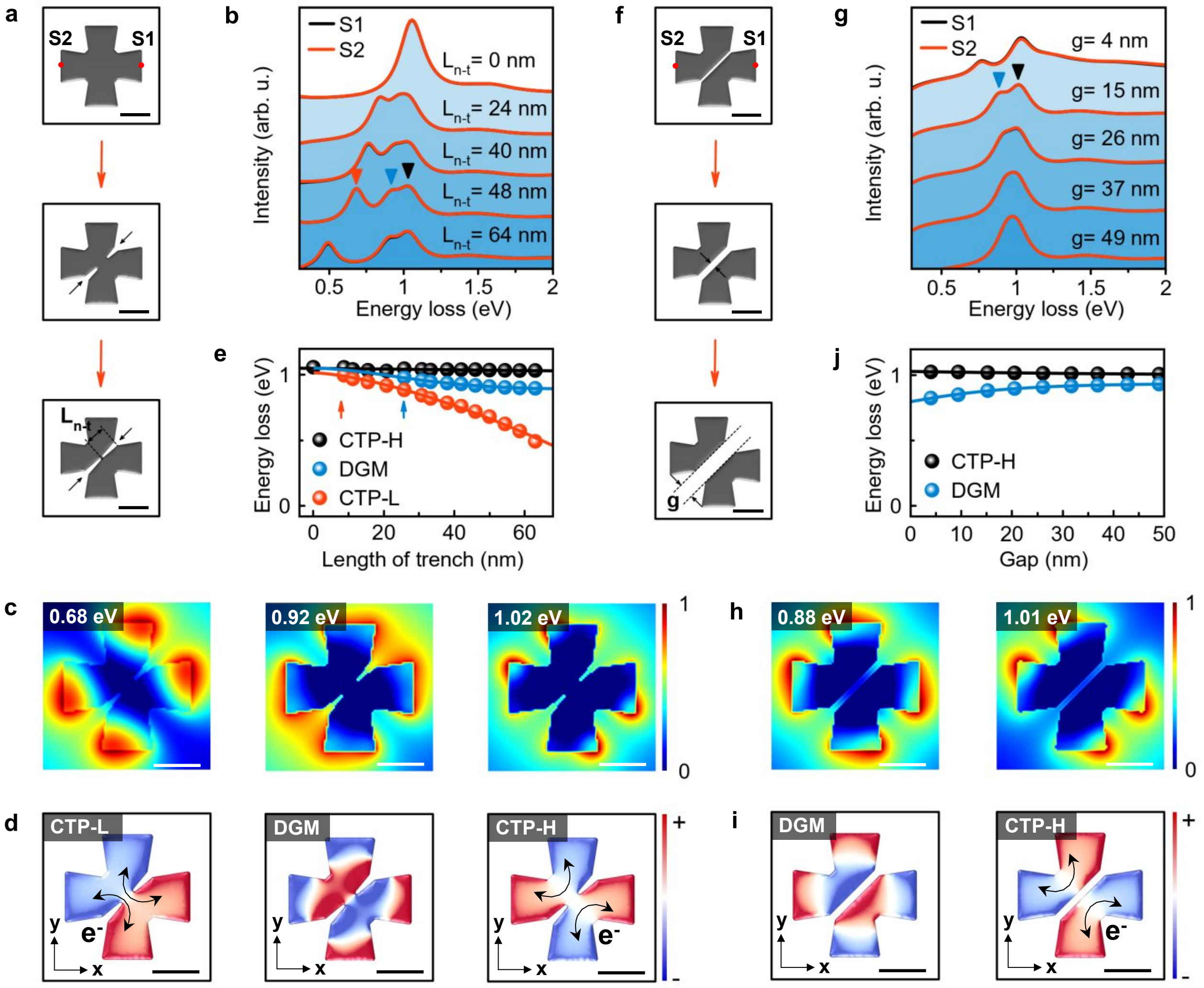

Figure S17. Tuning CTPs by DGM resonances. (a) Models showing Al nanocrosses without a nano-trench and with two nano-trenches in parallel. (b) Simulated EEL spectra for Al nanocrosses with different trench lengths. The spectra are derived from the locations marked with red dots on the models in (a). (c) Simulated EELS maps of an Al nanocross with a trench length of 48 nm. The EELS maps are extracted at different energies. (d) Computed eigenmodes corresponding to the plasmon modes observed in (c). (e) EEL spectra for Al nanocrosses with two nano-trenches in

parallel as a function of trench length. (f) Models showing an Al nanocross with different gap sizes. (g) Simulated EEL spectra for IAlNs with different gap sizes. (h) Simulated EELS maps of an Al nanocross with a gap size of 15 nm. The EELS maps are extracted at energies of 0.88 and 1.01 eV. (i) Calculated eigenmodes corresponding to the plasmon modes observed in (h). (j) EEL spectra for Al nanocrosses with different gap sizes. The scale bars are 100 nm (a,c,d,f,h,i).

Note S11. Identification of CTP-I resonance

Figure S18a,b displays an Al nanocross with a nano-trecnh having maximum trench width $W_{n\text{-}t}$ of 54 nm and the EEL spectra derived from the marked positions on the model. From the EELS maps and their corresponding eigenmodes, we notice the formation of a distinct third plasmon resonance of CTP-I between CTP-L and CTP-H resonances.

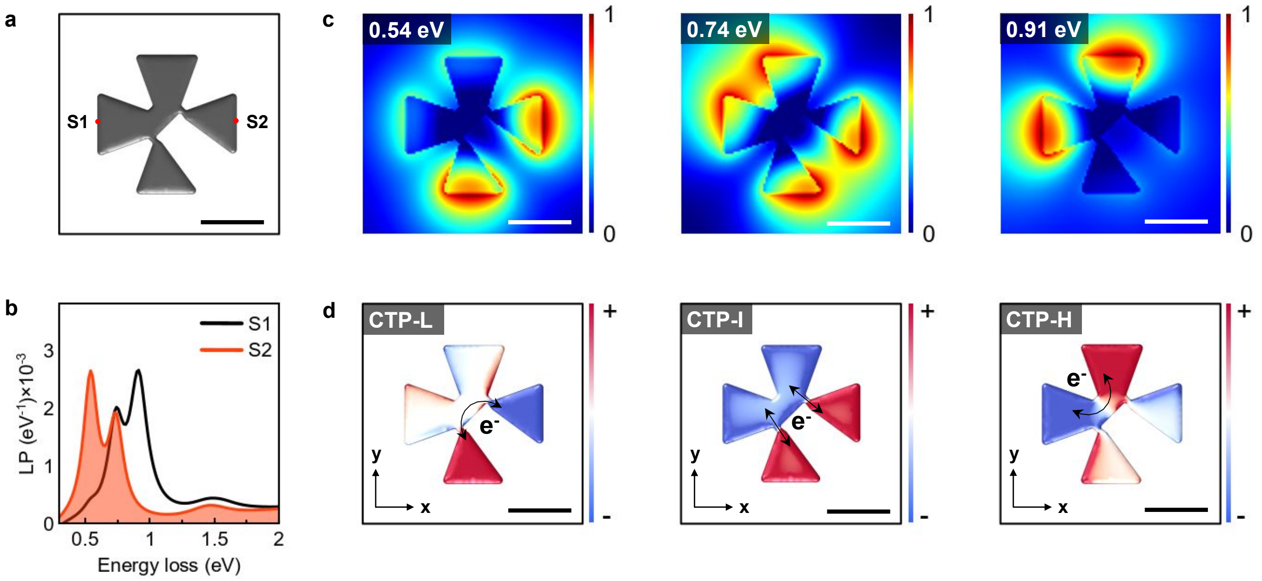

Figure S18. CTP modes of a trenched Al nanocross with a maximum trench width. (a) The model showing an Al nanocross with a nano-trench possessing a trench width of 54 nm. (b) EEL spectra derived from the positions marked with red dots on the model in (a). (c) Simulated EELS maps obtained at different energies. (d) Simulated eigenmodes corresponding to the plasmon resonances shown in (c). The scale bars are 100 nm (a,c,d).

Note S12. BEM simulations for Al nanocrosses with varying trench lengths

Figures S19a,b show the BEM simulations of EEL spectra for Al nanocrosses with two and four nanoprims, respectively. In both cases, the nanocross structures contain nanotrenches of different lengths. Note that when L= 0 nm, there are no nanotrenches present in either structure. As shown in Figure S19a,b, the CTP mode excited near the distorted edge of both Al nanocrosses exhibits a redshift as the trench length increases.

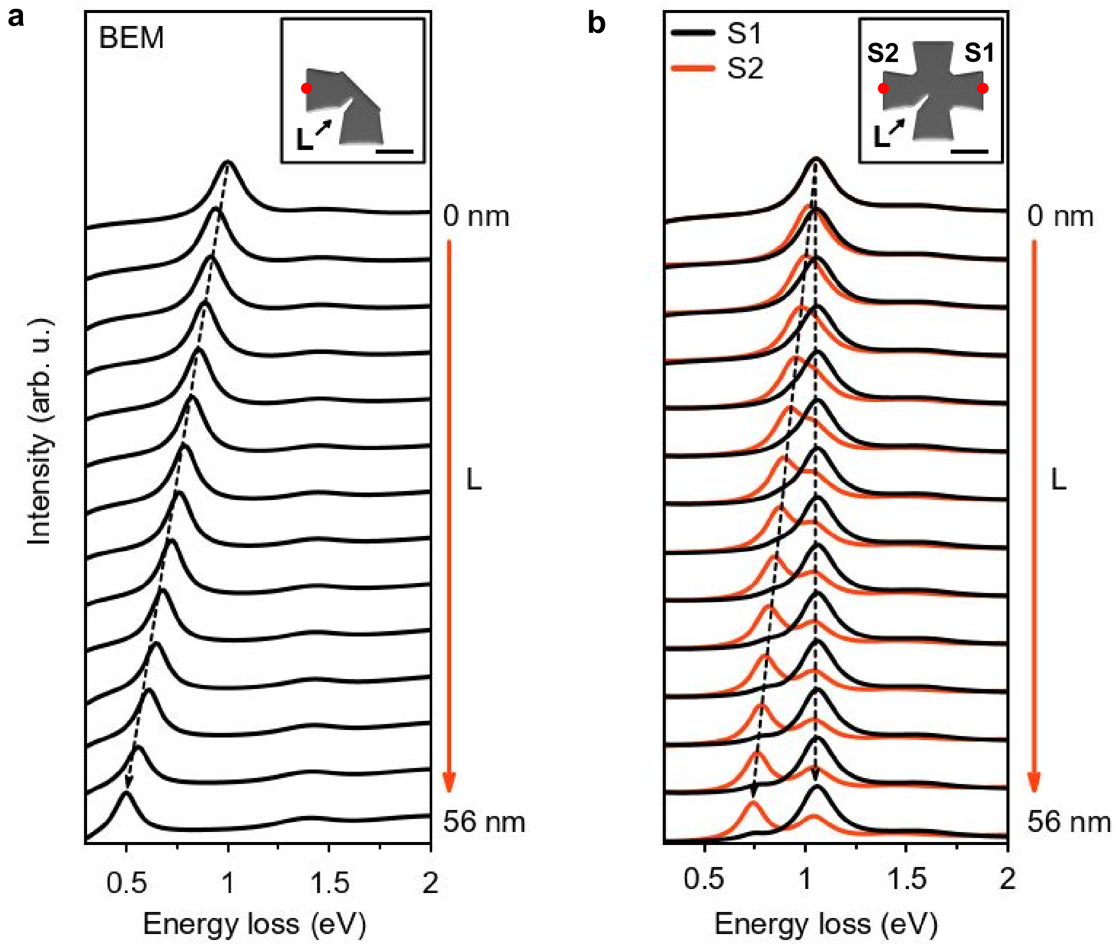

Figure S19. BEM simulations for Al nanocrosses with varying trench lengths. (a,b) Simulated EEL spectra derived from the marked positions on the models. The lengths of the nanotrenches are varied from 0 to 56 nm in (a) and (b). The scale bars are 100 nm (a,b).

Note S13. COM fitting to experimental EEL spectra

To demonstrate the agreement between the experimental EEL spectra and the COM simulations of the EEL spectra, fitting procedures were performed as shown in Figure S20. In the case of the Al nanocrosswithout a nanotrench, the EEL spectra show a close agreement to the COM for g=0 meV (Figure S20a,d). Upon the introduction of a nanotrench within the junction area of the same nanocross structure, the EEL spectra obtained from both edges of the nanocross show a good agreement with the COM for g=60 meV (Figure S20b,c,e,f).

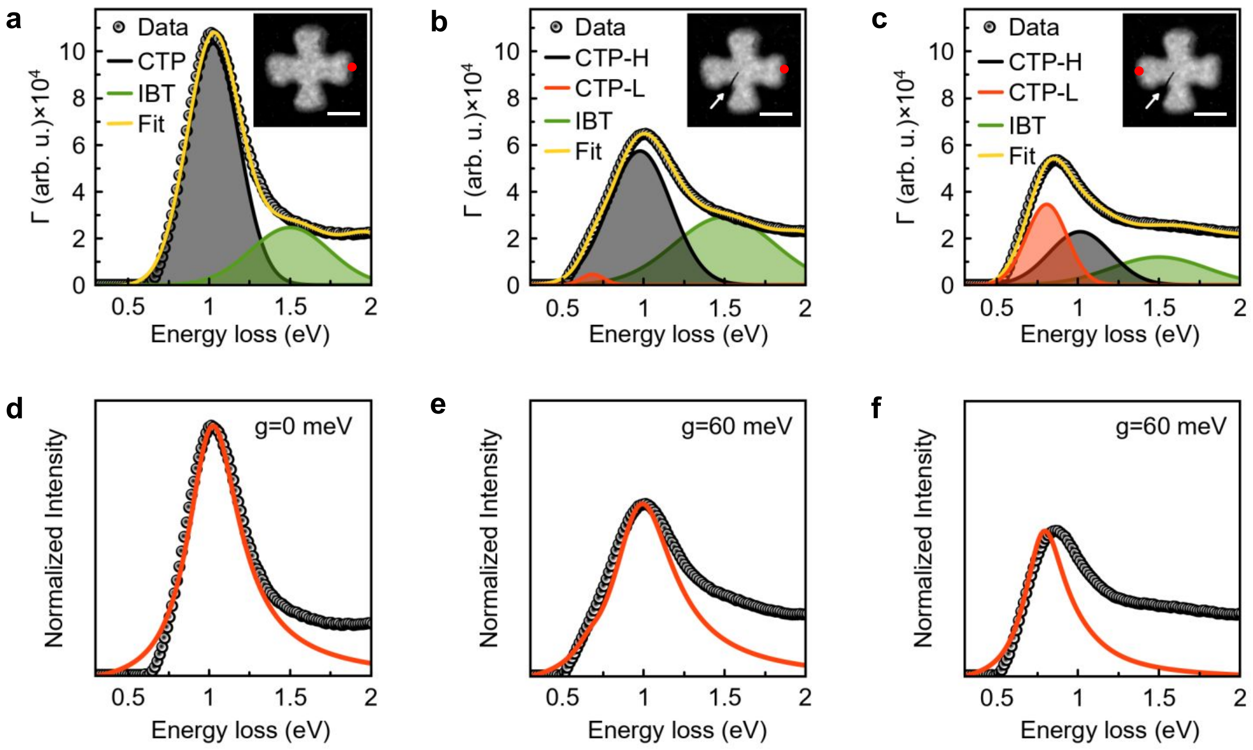

Figure S20. COM fitting to EEL spectra. (a) EEL spectra acquired at the red spot on the HAADF image of the Al nanocross without a nano-trench. (b,c) EEL spectra acquired at the red spots on the HAADF images of the same nanocross with a nano-trench. The EEL spectra are deconvolved using Gaussian curve fitting to extract the peak positions in (a-c). (d-f) COM fitting to the EEL spectra shown in (a-c), respectively. The scale bars are 100 nm (a,b,c).

Note S14. COM fitting to EEL spectra recorded on an Al nanocross with a larger junction

Figure S21 shows the COM fitting to both experimental and simulated EEL spectra obtained from another Al nanocross featuring a larger junction area. Both the experimental data (Figure S21a-c) and the simulated spectra (Figure S21d-f) show a good agreement with the COM for g=10 meV.

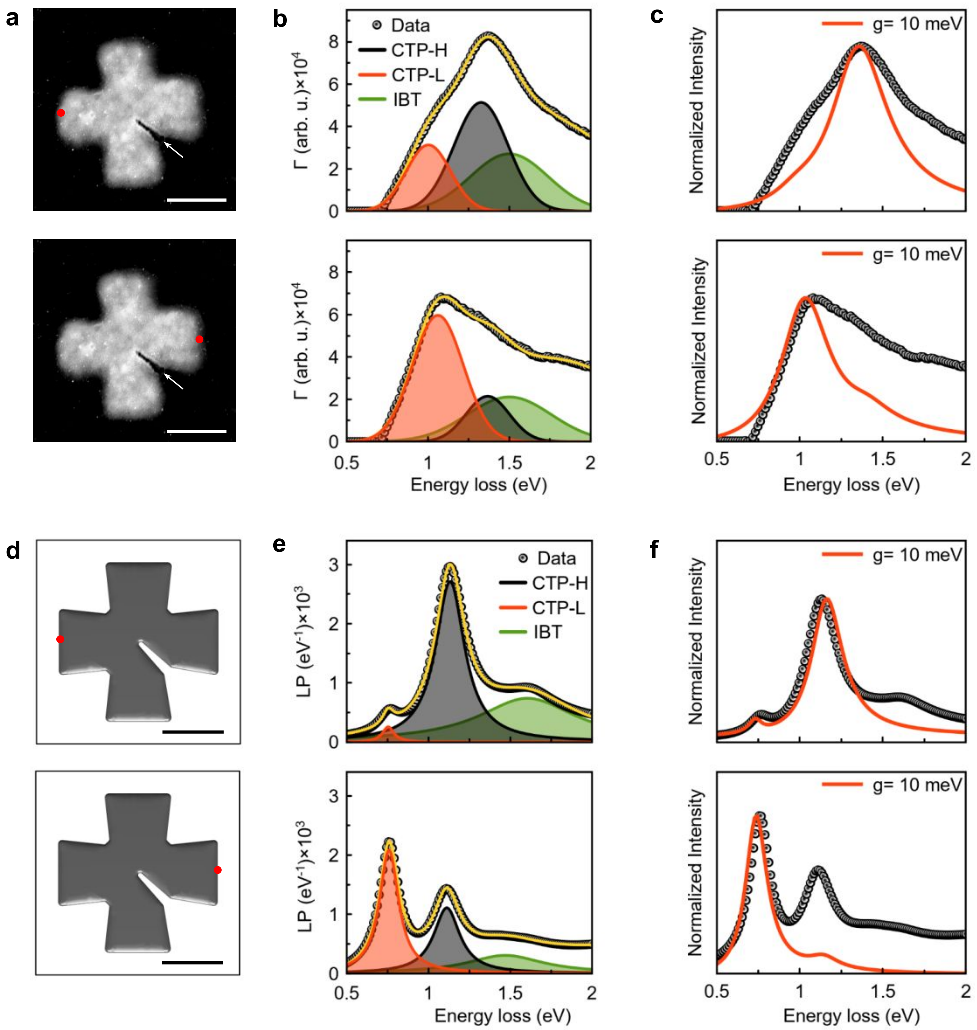

Figure S21. COM fitting to the EEL spectra shown in Figure S11e,f. (a) HAADF image of the Al nanocross with a nano-trench. (b) EEL spectra recorded at different positions marked with red spots on the HAADF images. EEL spectra are deconvoluted with Gaussian curve fitting to extract the peak positions. (c) COM fitting to the EEL spectra in (b). (d) Models showing the nanocross

corresponding to the experimental structure in (a). (e) EEL spectra derived at the different positions marked with red spots on the models in (d). EEL spectra are deconvoluted with Lorentzian curve fitting to extract the peak positions. (f) COM fitting to the simulated EEL spectra in (e). The scale bars are 100 nm (a,d).

Note S15. Energy resolution

The EEL spectra acquired in a vacuum environment show the energy resolutions of 0.17 eV (Figure S22).

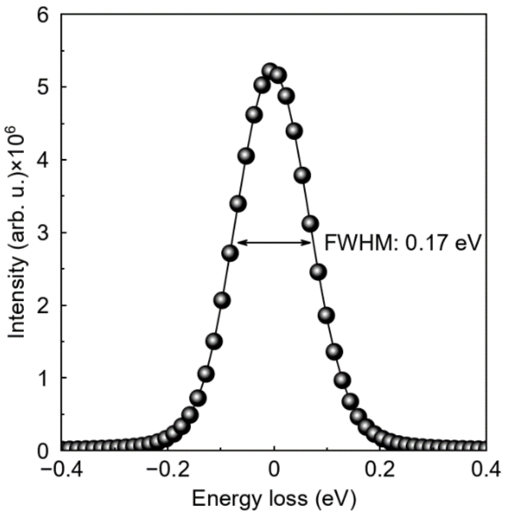

Figure S22. Energy resolution in low-loss EELS measurements. Zero-loss spectra recorded in the Zeiss SESAM microscope.

SUPPLEMENTARY REFERENCES

(1) Valamanesh, M.; Borensztein, Y.; Langlois, C.; Lacaze, E. Substrate Effect on the Plasmon Resonance of Supported Flat Silver Nanoparticles. *The Journal of Physical Chemistry C* **2011**, *115* (7), 2914-2922. DOI: 10.1021/jp1056495.

(2) Wiesner, M.; Roberts, R. H.; Lin, J.-F.; Akinwande, D.; Hesjedal, T.; Duffy, L. B.; Wang, S.; Song, Y.; Jenczyk, J.; Jurga, S.; et al. The effect of substrate and surface plasmons on symmetry breaking at the substrate interface of the topological insulator Bi2Te3. *Scientific Reports* **2019**, *9* (1), 6147. DOI: 10.1038/s41598-019-42598-9.

(3) Rasskazov, I. L.; Karpov, S. V.; Panasyuk, G. Y.; Markel, V. A. Overcoming the adverse effects of substrate on the waveguiding properties of plasmonic nanoparticle chains. *Journal of Applied Physics* **2016**, *119* (4). DOI: 10.1063/1.4940415 (acccessed 12/6/2023).

(4) Cherqui, C.; Li, G.; Busche, J. A.; Quillin, S. C.; Camden, J. P.; Masiello, D. J. Multipolar Nanocube Plasmon Mode-Mixing in Finite Substrates. *The Journal of Physical Chemistry Letters* **2018**, *9* (3), 504-512. DOI: 10.1021/acs.jpclett.7b03271.

(5) Maiti, A.; Maity, A.; Chini, T. K. Mode Mixing and Substrate Induced Effect on the Plasmonic Properties of an Isolated Decahedral Gold Nanoparticle. *The Journal of Physical Chemistry C* **2015**, *119* (32), 18537-18545. DOI: 10.1021/acs.jpcc.5b03686.

(6) Elibol, K.; van Aken, P. A. Uncovering the Evolution of Low-Energy Plasmons in Nanopatterned Aluminum Plasmonics on Graphene. *Nano Letters* **2022**, *22* (14), 5825-5831. DOI: 10.1021/acs.nanolett.2c01512.

(7) Elibol, K.; van Aken, P. A. Hybrid Graphene-Supported Aluminum Plasmonics. *ACS Nano* **2022**, *16* (8), 11931-11943. DOI: 10.1021/acsnano.2c01730.

(8) Elibol, K.; Mangler, C.; Gupta, T.; Zagler, G.; Eder, D.; Meyer, J. C.; Kotakoski, J.; Bayer, B. C. Process Pathway Controlled Evolution of Phase and Van-der-Waals Epitaxy in In/In2O3 on Graphene Heterostructures. *Advanced Functional Materials* **2020**, *30* (34), 2003300. DOI: https://doi.org/10.1002/adfm.202003300.

(9) Gupta, T.; Elibol, K.; Hummel, S.; Stöger-Pollach, M.; Mangler, C.; Habler, G.; Meyer, J. C.; Eder, D.; Bayer, B. C. Resolving few-layer antimonene/graphene heterostructures. *npj 2D Materials and Applications* **2021**, *5* (1), 53. DOI: 10.1038/s41699-021-00230-3.

(10) Polyushkin, D. K.; Milton, J.; Santandrea, S.; Russo, S.; Craciun, M. F.; Green, S. J.; Mahe, L.; Winolve, C. P.; Barnes, W. L. Graphene as a substrate for plasmonic nanoparticles. *Journal of Optics* **2013**, *15* (11), 114001. DOI: 10.1088/2040-8978/15/11/114001.

(11) Palanisamy, P.; Howe, J. M. Melting and supercooling studies in submicron Al particles using valence electron energy-loss spectroscopy in a transmission electron microscope. *Journal of Applied Physics* **2011**, *110* (2). DOI: 10.1063/1.3609063 (acccessed 9/19/2024).

(12) Zhu, D.; Bosman, M.; Yang, J. K. W. A circuit model for plasmonic resonators. *Optics Express* **2014**, *22* (8), 9809-9819. DOI: 10.1364/OE.22.009809.

(13) Huang, C.-p.; Yin, X.-g.; Huang, H.; Zhu, Y.-y. Study of plasmon resonance in a gold nanorod with an LC circuit model. *Optics Express* **2009**, *17* (8), 6407-6413. DOI: 10.1364/OE.17.006407.

(14) Wu, G.-j.; Zhang, Y.-h.; Huang, C.-p. Improved circuit model for plasmonic resonance of single split-ring resonators. *AIP Advances* **2022**, *12* (11). DOI: 10.1063/5.0125608 (acccessed 12/6/2023).

(15) Duan, H.; Fernández-Domínguez, A. I.; Bosman, M.; Maier, S. A.; Yang, J. K. W. Nanoplasmonics: Classical down to the Nanometer Scale. *Nano Letters* **2012**, *12* (3), 1683-1689. DOI: 10.1021/nl3001309.

(16) Elibol, K.; Downing, C.; Hobbs, R. G. Nanoscale mapping of shifts in dark plasmon modes in sub 10 nm aluminum nanoantennas. *Nanotechnology* **2022**, *33* (47), 475203. DOI: 10.1088/1361-6528/ac8812.

(17) Hobbs, R. G.; Manfrinato, V. R.; Yang, Y.; Goodman, S. A.; Zhang, L.; Stach, E. A.; Berggren, K. K. High-Energy Surface and Volume Plasmons in Nanopatterned Sub-10 nm Aluminum Nanostructures. *Nano Letters* **2016**, *16* (7), 4149-4157. DOI: 10.1021/acs.nanolett.6b01012.
(18) Knight, M. W.; King, N. S.; Liu, L.; Everitt, H. O.; Nordlander, P.; Halas, N. J. Aluminum for Plasmonics. *ACS Nano* **2014**, *8* (1), 834-840. DOI: 10.1021/nn405495q.
